# Synergistic gene editing in human iPS cells via cell cycle and DNA repair modulation

**DOI:** 10.1038/s41467-020-16643-5

**Published:** 2020-06-08

**Authors:** Thomas L. Maurissen, Knut Woltjen

**Affiliations:** 1 Graduate School of Medicine, Kyoto University, Kyoto, 606-8501 Japan; 2 Department of Life Science Frontiers, Center for iPS Cell Research and Application (CiRA), Kyoto University, Kyoto, 606-8507 Japan

**Keywords:** Genetic engineering, CRISPR-Cas9 genome editing, Induced pluripotent stem cells

## Abstract

Precise gene editing aims at generating single-nucleotide modifications to correct or model human disease. However, precision editing with nucleases such as CRIPSR-Cas9 has seen limited success due to poor efficiency and limited practicality. Here, we establish a fluorescent DNA repair assay in human induced pluripotent stem (iPS) cells to visualize and quantify the frequency of DNA repair outcomes during monoallelic and biallelic targeting. We found that modulating both DNA repair and cell cycle phase via defined culture conditions and small molecules synergistically enhanced the frequency of homology-directed repair (HDR). Notably, targeting in homozygous reporter cells results in high levels of editing with a vast majority of biallelic HDR outcomes. We then leverage efficient biallelic HDR with mixed ssODN repair templates to generate heterozygous mutations. Synergistic gene editing represents an effective strategy to generate precise genetic modifications in human iPS cells.

## Abstract (web summary)

Precision editing with CRISPR-Cas9 often suffers from poor efficiency. Here the authors identify culture conditions and small molecules that synergize to promote homology-directed repair (HDR) in induced pluripotent stem (iPS) cells.

## Introduction

Human induced pluripotent stem (iPS) cells are being widely employed to study human diseases, including inherited disorders, due to their ability to maintain a normal diploid karyotype through serial passages and differentiate into multiple derivative cell types. In general, genetic modeling consists of modifying a target site, either by deleting, inserting or replacing a specific DNA sequence^1^. More specifically, precision gene editing aims to make modifications in the genome to correct or recreate pathogenic variants at single-nucleotide resolution^2^. The CRISPR-Cas9 system is the most used tool to generate targeted DNA double strand breaks (DSBs) in the genome^3,4^, which are subsequently resolved by endogenous cellular DNA DSB repair pathways. Predominantly, non-homologous end joining (NHEJ) results in insertion and deletion (indel) mutations, while microhomology-mediated end joining (MMEJ) makes predictable deletions^5^. NHEJ and MMEJ are referred to collectively as mutagenic end joining (MutEJ) as both repair outcomes can lead to a loss or gain of DNA sequence. In order to generate single-nucleotide changes, the homology-directed repair (HDR) pathway is leveraged in combination with a customized repair template such as a donor plasmid or single-stranded donor oligonucleotide (ssODN)^6–9^. However, recent approaches have met limited efficiency and applicability that are largely due to a lack of direct selection^10^ and the predominance of MutEJ outcomes over precise repair^11,12^. Base editors combining deaminases with Cas9 nickase present a strategy to generate single-nucleotide substitutions with minimal MutEJ, but show restricted editing to a fixed 5-bp window and high risk for bystander editing, as well as a limited range and number of possible nucleotide substitutions^13^ and risk for off-target deaminase activity^14^. Thus, HDR editing with custom ssODN templates warrants further optimization.

Low HDR/MutEJ ratios^15–17^ have spurred efforts to improve precision editing by influencing DNA repair. Approaches shown to improve HDR rates include the modulation of DNA repair components by targeted NHEJ inhibition^18–21^, ectopic expression of HDR factors^22–24^ or the direct fusion of HDR-promoting proteins to Cas9^25–27^, and conjugation of ssODN repair templates to Cas9^28,29^. Inducing cold shock by a moderate reduction in incubation culture temperature was shown to increase the protein half-life and targeting efficiency of zinc finger nucleases (ZFN) and transcription activator-like effector (TALE)-nuclease chimeras (TALENs) in K562 and HeLa cells^30,31^ and more recently to improve HDR rates using Cas9 in iPS cells^32,33^, although the mechanisms in iPS cells remain unknown.

DNA repair pathway choice is closely tied to the cell cycle to safeguard genomic integrity during mitosis, and cell-cycle synchronization has been linked to improved HDR rates in various cell lines^34–36^. HDR is limited to the S and G2 phase, concurrent with the availability of homologous sequence that is used as a repair template during genomic insult^37,38^. On the other hand, NHEJ is active throughout the cell cycle^39^. Individually, strategies aimed to either control DNA repair, cell-cycle progression, or the availability of nuclease reagents and homologous repair templates have resulted in improved HDR rates. Yet despite continued efforts, no clear evidence of synergistic effects on gene editing efficiency has been reported to date^35,40,41^.

In this study, we show synergy between DNA DSB repair and the cell cycle in favoring the generation of ssODN-mediated single-nucleotide changes. Establishing a fluorescent DNA DSB repair assay based on GFP to BFP conversion^42^ in iPS cells, we screen compounds and culture conditions to evaluate their effects on DNA repair outcomes at the single-cell level by fluorescence-activated cell sorting (FACS) analysis. HDR rates and HDR/MutEJ ratios are improved when modulating DNA repair pathway choice by single or dual inhibition, and HDR rates are further improved by modulating cell-cycle progression with cold shock or pharmacological inhibition. However, high-frequency HDR editing predominantly leads to the generation of homozygous mutants in a biallelic reporter system. In order to generate heterozygous mutants under these conditions, we employ a strategy using mixed ssODN repair templates to protect one allele with a silent mutation. We then apply synergistic gene editing at endogenous autosomal loci and improve several-fold the precise generation of heterozygous and homozygous mutations compared to baseline HDR levels. These results show that controlling cell-cycle progression and DNA repair synergistically improve ssODN-mediated gene editing, as demonstrated through the generation of various isogenic models of genetic disease.

## Results

### Fluorescent DNA repair assay in human iPS cells

**Fig. 1:**
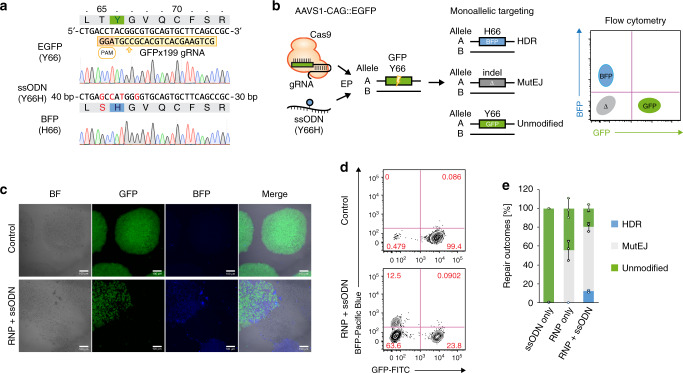
Visualization and quantification of DNA repair outcomes in a fluorescent DNA repair assay in human iPS cells. **a** Sequence of the GFP reporter target site and engineered modification to BFP. The sequence of gRNA GFPx199 is boxed in yellow, the PAM sequence in orange, the GFP fluorophore residue p.Y66 in green and BFP fluorophore residue p.H66 in blue. Nucleotide changes are indicated in red in the ssODN sequence and in the resulting BFP sequence product. **b** Schematic of the predicted outcomes from GFP editing in a heterozygous AAVS1-CAG::EGFP (GFP) iPS cell line, and their distribution in FACS. **c** Confocal microscopy images of the unedited heterozygous GFP iPS cell line (Control), and fluorescent conversion outcomes following co-transfection of Cas9 RNP and Y66H ssODN repair template (RNP + ssODN). Scalebars are 100 µm. Representative images of three independent experiments with similar results are shown. **d** Representative FACS plots of unedited GFP iPS cells (top) and cells treated with Cas9 RNP and Y66H ssODN (bottom). **e** Quantification of HDR, MutEJ or unmodified DNA repair outcomes by FACS analysis. Data are presented as the mean ± S.D. of three technical replicates of independent electroporations for each respective condition. Source data are provided as a Source Data file.

Fluorescent reporters provide a means to visualize and quantify gene editing outcomes. GFP and BFP share high sequence homology, and a single tyrosine to histidine (Y66H) amino-acid change in the fluorophore region is sufficient to convert GFP to BFP fluorescence emission^42^. We generated an iPS cell line heterozygously targeted at the AAVS1 locus in the 1383D6 parental background (Supplementary Fig. 1) with a stably expressing GFP fluorescent reporter following our previously published method^43^. A Cas9-mediated DSB in GFP was repaired using a 100 bp ssODN template designed to make four nucleotide changes resulting in conversion of GFP to BFP (Y66H), insertion of a PAM blocking mutation to prevent Cas9 recleavage and stabilize the BFP protein (T65S), and creation of a *Nco*I restriction site for genotyping by restriction fragment length polymorphism (RFLP) (Fig. 1a). Three possible fluorescent outcomes reflect DNA repair choices: BFP, loss of fluorescence (Δ), and GFP, corresponding to HDR, MutEJ, and unmodified alleles, respectively (Fig. 1b). Due to uniform expression of the reporter from the AAVS1 locus, these three outcomes could be clearly discriminated and quantified following nuclease treatment (Fig. 1c–e; Supplementary Fig. 2).

### Cell-cycle control favors HDR outcomes

**Fig. 2:**
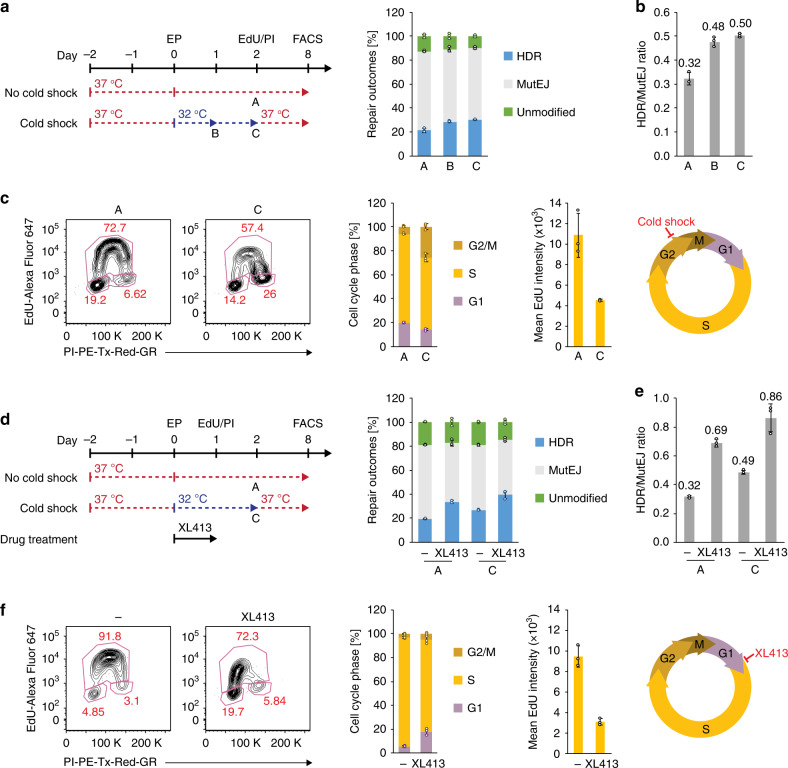
Cold shock and cell-cycle synchronization with XL413 improve HDR efficiency in iPS cells. **a** Experimental timeline with no cold shock [A], and cold shock for 24 h [B] or 48 h [C] following electroporation (EP) on day 0 (left). EdU/PI staining was performed after 48 h of culture, and FACS analysis on day 8. The resulting effect on DNA repair outcome frequency is shown (right). **b** Ratio of HDR/MutEJ repair outcomes measured in **a**. **c** Representative FACS plots of EdU/PI staining (left) for quantification of cell-cycle phase (middle left) and mean EdU intensity of S-phase cells (middle right) 48 h after EP with no cold shock [A] or cold shock for 48 h [C] following EP. Schematic of the cell cycle showing cold shock arrest in G2/M phase (right). **d** Experimental timeline (left) of XL413-induced cell-cycle arrest for 24 h post-EP, in the absence [A] or presence [C] of cold shock. EdU/PI staining was performed after 24 h of culture, and FACS analysis on day 8. Repair outcomes were quantified in the absence or presence of XL413 (right). **e** Ratio of HDR/MutEJ repair outcomes measured in **d**. **f** Representative FACS plots of EdU/PI staining (left) 24 h after EP for untreated and XL413-treated cells under normal culture conditions [A], and quantification of cell-cycle phase (middle left) and mean EdU intensity of S-phase cells (middle right). Schematic of XL413-induced cell-cycle arrest in the G1/early S phase (right). All data are presented as the mean ± S.D. of three technical replicates for each respective treatment. Source data are provided as a Source Data file.

Considering the link between cell-cycle phase and DNA repair pathway choice, we aimed to synchronize cells using various culture conditions and chemical inhibitors. Mild hypothermia has been shown to cause cell-cycle arrest and slow cell metabolism in vitro^44^. We observed that applying a 32 °C cold shock for 48 h directly following electroporation (EP) improved HDR frequency 1.4-fold (30.1% vs 21.3%) and the HDR/MutEJ ratio 1.6-fold compared to normal culture conditions at 37 °C (Fig. 2a, b; Supplementary Fig. 3a, b), confirming previously published results^32,33^.

In order to elucidate the effect of cold shock on iPS cells, we performed cell-cycle analysis using a 5-ethynyl-2′-deoxyuridine (EdU) proliferation assay combined with propidium iodide (PI) staining for total DNA content, and quantified the cell-cycle phase after EP and cold shock treatment (Fig. 2c). Cells undergoing cold shock for 48 h after EP showed 25.5% of cell-cycle phase accumulation in the G2/M phase compared to 6.0% under normal culture conditions. In addition, the mean intensity of EdU incorporation was reduced 2.4-fold in S-phase cells in the cold shock condition, indicating a reduced DNA synthesis rate. We tested the effect of cell-cycle synchronization in late G2/M phase by pretreating cells with Nocodazole for 16 h before EP but, in disagreement to previous reports^34,35^, we observed no increase in HDR efficiency (Supplementary Fig. 3c–e). This might suggest that slowed cell-cycle progression in the presence of nuclease is more important than G2/M synchronization prenuclease. Nocodazole inhibits microtubule polymerization, activating the spindle assembly checkpoint and arresting cells in prometaphase, likely causing cells to immediately complete mitosis upon nocodazole release.

Since HDR is most active in S/G2 phase, we investigated the effect of synchronizing cells using the CDC7 inhibitor XL413, which halts S-phase progression and arrests karyotypically normal cells in G1/S^45,46^. As expected, EdU/PI staining revealed accumulation of >90% of cells in G1 and early S phase after 24 h treatment with XL413 after EP (Fig. 2d–f). XL413 increased the HDR frequency 1.7-fold (33.7% vs 19.4%), and the HDR/MutEJ ratio 2.2-fold compared to the untreated control. When combining XL413 treatment for 24 h with cold shock for 48 h, the HDR frequency was increased further by 2-fold (39.4% vs 19.4%) and the HDR/MutEJ ratio by 2.7-fold (0.86 vs 0.32), such that HDR events became nearly as frequent as MutEJ. Interestingly, pretreatment of cells with XL413 before EP did not improve HDR (Supplementary Fig. 3f–h). These results indicate that chemical and environmental factors modulating cell-cycle synchronization and progression can individually and synergistically affect DNA repair pathway choice in favor of higher HDR outcomes.

### Modulating DNA repair pathways to bias HDR outcomes

**Fig. 3:**
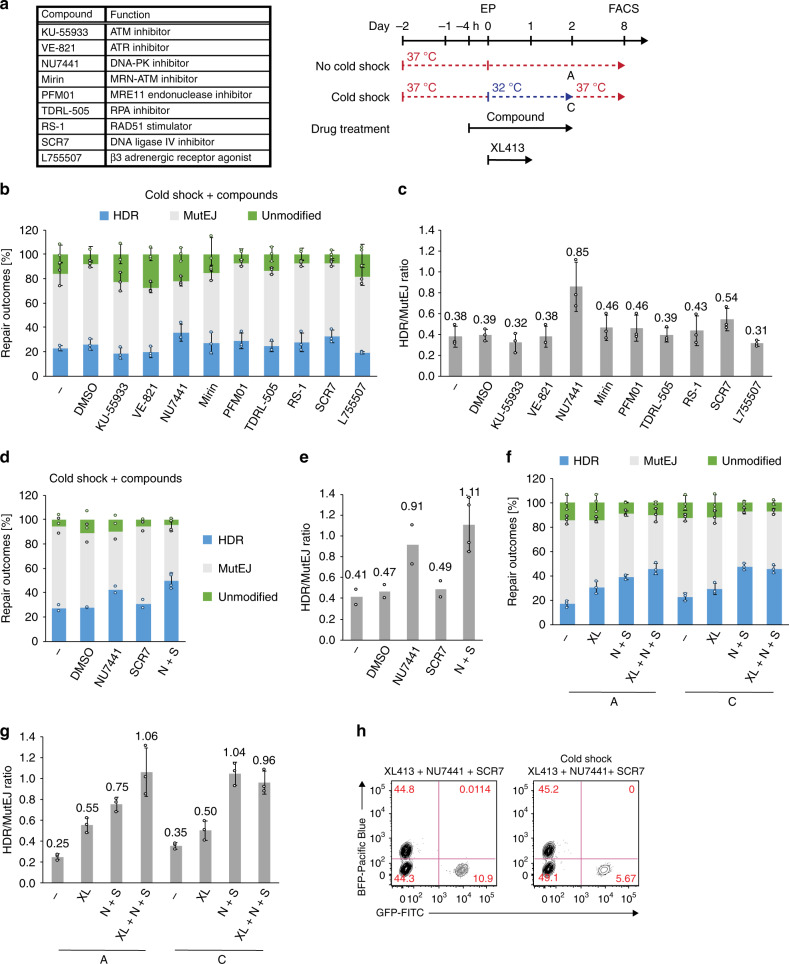
Combining DNA repair modulation and cell-cycle synchronization synergistically enhances HDR in iPS cells. **a** List of compounds tested with their function on molecular targets (top), and experimental timeline of compound pre- (4 h) and post-EP (48 h) treatment (bottom). **b** Screening of compounds under cold shock condition [C], including untreated (−) and DMSO treated (DMSO) controls, showing the effect on DNA repair outcome frequency. Data are presented as the mean ± S.D. of three biological replicates. **c** Ratio of HDR/MutEJ repair outcomes measured in **b**. **d** Combination treatment of NU7441 and SCR7 under cold shock condition [C], and the effect on repair outcome frequency. Data are presented as the mean of two biological replicates, except N + S is presented as the mean ± S.D. of four biological replicates. **e** Ratio of HDR/MutEJ repair outcomes measured in **d**. **f** Combination treatment of cell-cycle inhibitor XL413 (XL) post-EP (24 h) and N + S pre- (4 h) and post-EP (48 h) under normal culture conditions [A] or cold shock [C]. Data are presented as the mean ± S.D. of three technical replicates for each respective treatment. **g** Ratio of HDR/MutEJ repair outcomes measured in **f**. **h** Representative FACS plots of cells treated with XL413 (XL) and N + S under normal [A] (left) and cold shock [C] (right) conditions, as tested in **f**. Source data are provided as a Source Data file.

To further improve precise gene editing, we treated cells with various small molecules (Fig. 3a) reported to inhibit particular DNA repair pathway components: KU-55933^47^, VE-821^48^, NU7441^49^, Mirin^50^, PFM01^51^, TDRL-505^52^, and SCR7^53^; or enhance HDR rates: RS-1^54^ and L755507^55^. Human iPS cells were treated with small molecules for 4 h before and 48 h following EP, in addition to 48 h cold shock. Among screened compounds, NU7441 (a DNA-PKcs inhibitor) and SCR7 (a ligase IV inhibitor) increased the HDR frequency 1.6-fold and 1.4-fold (35.5% and 32.3% vs 22.6%; Fig. 3b) and were the only two compounds with an HDR/MutEJ ratio > 0.5. Interestingly, compared to NU7441, SCR7-treated samples also showed a larger fraction of MutEJ, resulting in a reduced HDR/MutEJ ratio increase (1.4-fold vs 2.2-fold for NU7441) despite similar proportions of HDR (Fig. 3c). When combined, NU7441 + SCR7 (N + S) had a cumulative effect further improving HDR frequency by 1.8-fold (49.6% vs 27.3%) and the HDR/MutEJ ratio by 2.7-fold compared to untreated control (1.11 vs 0.41), resulting in a majority of HDR events (Fig. 3d, e). On the other hand, previously reported HDR enhancers such as RS-1 and L755507 showed only a modest change in HDR frequency. These data suggest that inhibiting several molecular targets within the NHEJ repair pathway can have an additive effect, further promoting the use of HDR for DSB repair.

We next combined chemical cell-cycle synchronization with XL413 and NHEJ repression with N + S treatment, in order to verify possible additive effects improving HDR rates (Fig. 3f–h). Under normal culture conditions, treatment with XL413 (XL) or N + S alone increased HDR frequency by 1.8-fold and 2.3-fold (30.5 and 39% vs 17%), respectively, while combining XL413 with N + S further increased the HDR frequency by 2.7-fold (45.7% vs 17%) compared to an untreated control. Under cold-shock conditions, comparable changes in HDR frequency were observed for individual treatments, but the combination of XL413 with N + S did not show improvement in HDR frequency compared to N + S treatment alone. In brief, we show evidence for the existence of synergistic gene editing enhancing HDR outcome frequencies when combining modulation of DNA repair with cold shock, or with cell-cycle synchronization in G1/S phase.

### Highly efficient biallelic gene editing

**Fig. 4:**
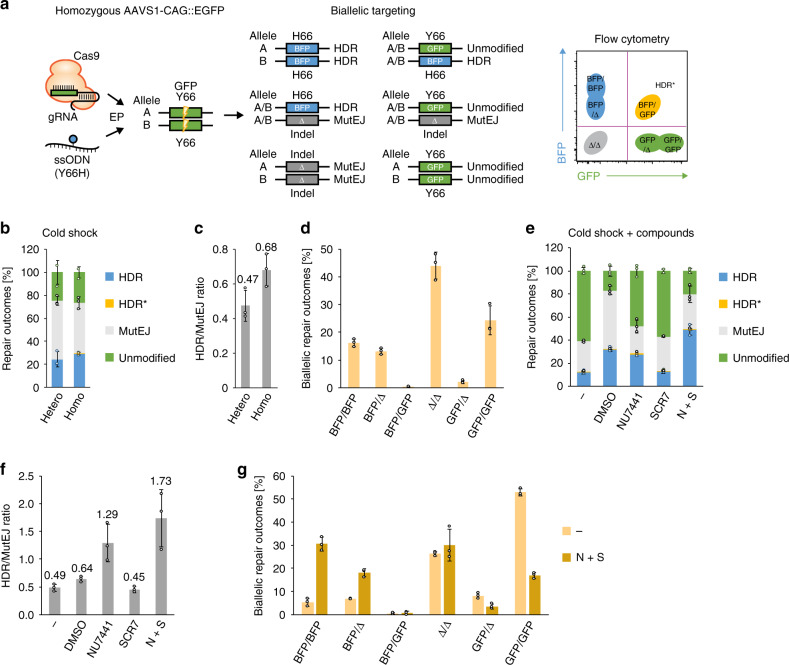
Homozygous fluorescent DNA repair assay to visualize and quantify allele-specific DNA repair outcomes during biallelic editing in iPS cells. **a** Schematic of the predicted outcomes from GFP editing in a homozygous AAVS1-CAG::EGFP (GFP) iPS cell line, and their distribution in FACS. **b** Comparison of monoallelic and biallelic editing in heterozygous and homozygous GFP iPS cells, showing DNA repair outcome frequency. HDR* indicates double-positive BFP/GFP cells arising from heterozygous editing and HDR repair in homozygous GFP iPS cells. **c** Ratio of HDR/MutEJ repair outcomes measured in **b**. **d** Quantification of biallelic repair outcomes in homozygous GFP iPS cells obtained from FACS-gating on each possible DNA repair outcome represented in **a**. **e** Effect of combined cold shock and drug treatment on repair outcome frequency in homozygous GFP iPS cells. **f** Ratio of HDR/MutEJ repair outcomes measured in **e**. **g** Biallelic repair outcomes in the absence (−) or presence (N + S) of combined drug treatment obtained from DNA repair outcomes represented in **e**. All data are presented as the mean ± S.D. of three technical replicates for each respective treatment. Source data are provided as a Source Data file.

In diploid iPS cells, both paternal and maternal alleles are candidates for editing. In order to recapitulate this scenario, we generated a homozygous GFP reporter iPS cell line in the 1383D6 parental background (Supplementary Fig. 1) and evaluated biallelic gene targeting outcomes (Fig. 4a). We first compared monoallelic and biallelic targeting efficiencies and DNA repair outcome frequencies between heterozygous and homozygous GFP reporter lines (Fig. 4b) and found comparable HDR frequencies in both lines (24.3% and 29.2%). In the homozygous context, we gated cells having at least one BFP allele as HDR outcomes, and cells having at least one GFP allele as unmodified, which results in a reduced fraction of MutEJ cells and a 1.4-fold higher HDR/MutEJ ratio compared to the heterozygous cell line (Fig. 4c). Surprisingly, there was no detectable double-positive population that represents heterozygote mutants bearing one HDR allele (BFP) and one unmodified allele (GFP), referred to as HDR* (Fig. 4a). To further explore the cause, we deconvolved all possible biallelic repair outcomes to estimate individual allele repair efficiencies (Fig. 4d). Here, we obtained 16.1% BFP/BFP precise biallelic editing, and a predominant fraction of MutEJ alleles distributed across BFP/Δ (13.1%), Δ/Δ (44%), and GFP/Δ (2.1%) repair outcomes. In summary, this data revealed that both alleles are mostly coedited or unmodified, as shown by the low frequency of monoallelic editing in GFP/Δ (2.1%) and BFP/GFP (0.3%) repair outcomes.

We further applied NU7441, SCR7, and N + S under cold shock treatment to the homozygous GFP line and observed a 4-fold increase in HDR frequency (48.9% vs 12.2%) and a 3.7-fold increase in HDR/MutEJ ratio (1.73 vs 0.49) compared to untreated control (Fig. 4e, f). Deconvolution of biallelic repair outcomes showed 30.5% BFP/BFP biallelic repair outcomes when using N + S treatment, 5.7-fold higher than the untreated condition (5.3%) (Fig. 4g). These results demonstrated high biallelic targeting efficiency when combining cold shock treatment and DNA repair pathway inhibitors, leading to the effective generation of homozygous mutants containing single-nucleotide modifications. Interestingly, under the conditions we tested, the proportion of heterozygous BFP/GFP outcomes was consistently low (0.7% and 0.5% with and without N + S treatment) indicating that although homozygous outcomes are frequent, an alternative targeting strategy would be required to efficiently generate heterozygous mutations.

### Generation of heterozygous mutations

**Fig. 5:**
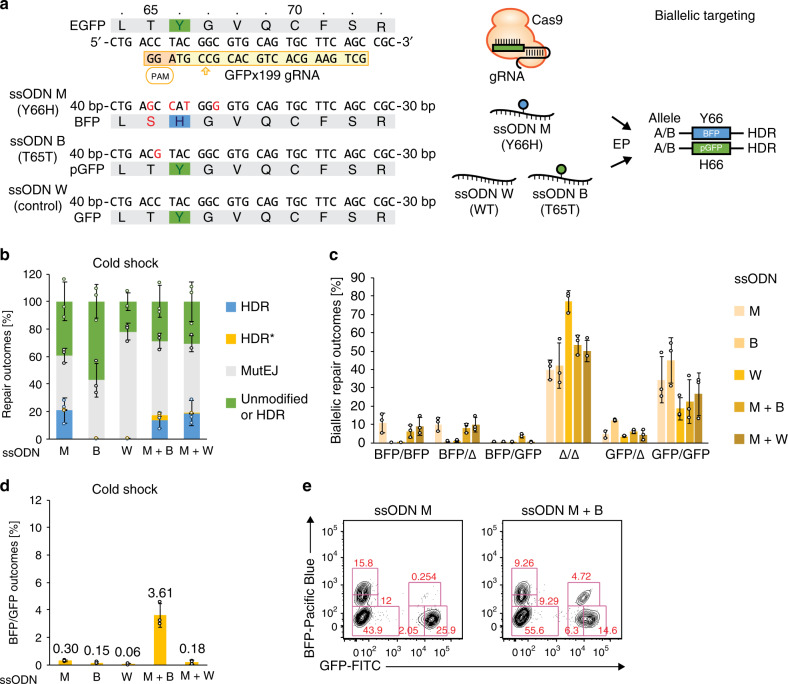
Generation of heterozygous mutations requires protection of one allele. **a** Target sequence in the GFP reporter, and ssODN repair templates to create a missense Y66H mutation (ssODN M), a silent T65T blocking mutation (ssODN B) or no mutation (ssODN W). HDR repair of ssODN B T65T results in a protected GFP (pGFP) allele. Cells bearing unmodified or pGFP alleles are indistinguishable by FACS and are scored cumulatively as GFP. **b** GFP editing with ssODN M, B or W individually, or in M + B and M + W combination, and effect on DNA repair outcome frequency under cold shock condition. HDR* indicates the frequency of heterozygous double-positive BFP/GFP or compound heterozygous double-positive BFP/pGFP repair outcomes. GFP-positive cells include unmodified cells and HDR-mediated monoallelic pGFP/GFP, pGFP/indel (Δ), or biallelic pGFP/pGFP repair outcomes. **c** Distribution of biallelic repair outcomes shown in **b**. **d** Biallelic BFP/GFP repair outcome frequency measured in **c**. **e** Representative FACS plots of BFP conversion only (ssODN M) or heterozygous compound BFP/pGFP mutations (ssODN M + B). All data are presented as the mean ± S.D. of three technical replicates of independent electroporations for each respective condition. Source data are provided as a Source Data file.

In order to resolve low monoallelic editing rates while retaining high overall HDR frequencies, we adopted a mixed ssODN repair template strategy. Herein, we leveraged high biallelic modification rates to generate HDR-mediated compound heterozygous mutations composed of one edited allele and one protected allele bearing a silent blocking mutation, thereby preventing subsequent Cas9 recleavage and indel formation at the target site (Fig. 5a). Along with the Y66H-mutant ssODN (ssODN ‘M’), we designed an ssODN carrying a silent T65T blocking mutation (ssODN ‘B’), and a wild-type (WT) ssODN identical to the GFP target sequence (ssODN ‘W’) as a control. HDR-mediated editing of T65T with ssODN B destroys the PAM sequence at the target site, resulting in a Cas9-protected GFP allele (pGFP) (Supplementary Fig. 4a, b). Alleles incorporating ssODN W were expected to be susceptible to recleavage. Biallelic HDR repair outcomes were expected to result in the generation of homozygous BFP/BFP and pGFP/pGFP cells, as well as a fraction of desired compound heterozygous BFP/pGFP cells referred to as HDR*.

Single ssODN templates and ssODN combinations were tested (Fig. 5b, c). ssODN B showed a 1.5-fold increase in GFP-positive cells (57.2%) suggesting the generation of pGFP alleles by HDR, while ssODN W showed a 1.8-fold decrease (22.2%) compared to ssODN M (39.1%), suggesting that WT GFP alleles generated through HDR are subject to recleavage as predicted. Combinations of ssODN M + B and ssODN M + W yielded similar total repair frequencies. Nonetheless, among all ssODN conditions only the combination of ssODN M and B resulted in a population of heterozygous BFP/GFP double-positive cells, with an efficiency of 3.6% (Fig. 5d, e), 12-fold higher than using ssODN M alone (0.3%) and 20-fold higher than using a combination of ssODN M and W (0.18%). When applying N + S treatment in addition to cold shock, the efficiency of compound heterozygous BFP/pGFP generation retained a similar fold-enrichment over ssODN M (12.4-fold) or ssODN M + W (16.2-fold) alone but increased to 11.2% of the total population (Supplementary Fig. 4c–f). These results suggest that under conditions of high HDR efficiency, protection of one allele against Cas9-mediated recleavage is required in order to obtain heterozygous mutant alleles without a mutagenic indel in the second allele.

Consequently, we verified the applicability of this approach to endogenous loci. First, we targeted *ATP1A1* (Supplementary Fig. 5a) that is known to cause 2000-fold increase in dominant cellular resistance to the cytotoxic inhibitor ouabain when introducing Q118R and N129D missense mutations compared to making in-frame indel mutations^56^. Selecting for HDR clones under high ouabain concentration, we observed a 1.8-fold increase in colony number with combined cold shock and N + S treatment, indicating synergistic increase in frequency at an endogenous locus (Supplementary Fig. 5b). Next, we aimed to edit nonselectable endogenous loci. We independently generated the N588K (c.1764C > A) mutation in *KCNH2* and G201V (c.602 G > T) mutation in *PSMB8*, either using an ssODN carrying the mutation (ssODN M) or a combination of ssODN M and an ssODN carrying a silent blocking mutation (ssODN M + B) and performed clonal analysis (Supplementary Fig. 5c, d). Under optimal cold shock and drug treatment conditions, we obtained 14 out of 96 homozygous clones edited at *KCNH2* and 18 out of 91 at *PSMB8* when using KCNH2 or PSMB8 ssODN M only, corresponding to biallelic HDR events (Supplementary Fig. 5e, f). Moreover, we could only obtain compound heterozygous clones at KCNH2 (4/92) or PSMB8 (4/95) when using the ssODN M + B combination, corresponding to biallelic HDR events incorporating mutant ssODN M and silent blocking ssODN B at cognate alleles. These results confirm that our approach is highly effective to generate both homozygous and heterozygous clones at endogenous loci in human iPS cells.

### Synergistic gene editing enhances HDR at endogenous loci

Finally, in considering the application of gene-edited iPS cells for cell therapy, we tested our defined conditions using a transfection instrument approved for GMP cell applications. We compared DNA repair outcome frequencies in normal culture, cold shock, and combined cold shock and N + S conditions in heterozygous and homozygous GFP iPS cell lines generated in two different donor genetic backgrounds (Supplementary Fig. 6). In the 1383D6 genetic background, HDR efficiency increased 1.2-fold with cold shock and 1.6-fold with combined cold shock and N + S treatment both in heterozygous (59.1% and 75.6% vs 47.9%) and homozygous (64.6% and 84.1% vs 52.9%) cell lines compared to an untreated control. When editing homozygous GFP iPS cells with ssODN M and B, the efficiency of compound heterozygous BFP/pGFP editing increased by 1.5-fold with cold shock and 2.5-fold with combined cold shock and N + S treatment (14.4% to 24.1% vs 9.8%). Similar results were obtained in the 409B2 genetic background.

**Fig. 6:**
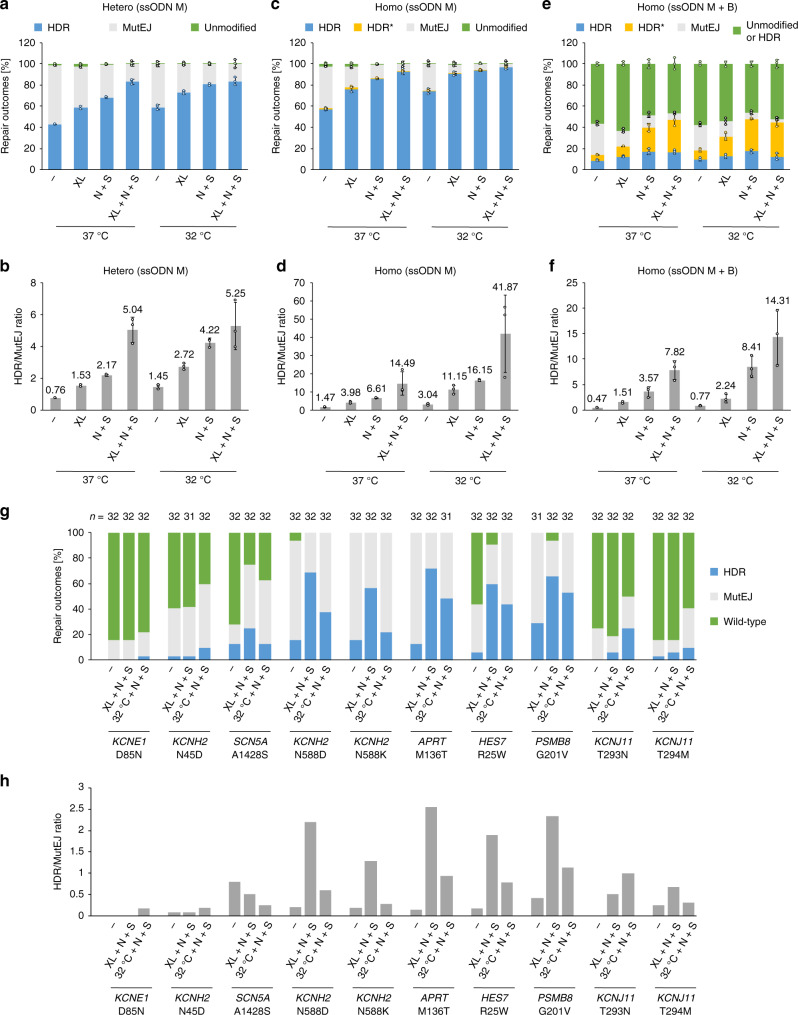
Synergistic gene editing at endogenous loci. **a** DNA repair outcomes of heterozygous (Hetero) GFP iPS cells generated in the 409B2 genetic background. Cells were targeted with Y66H-mutant ssODN M only (ssODN M), and treated individually or in combination with cell-cycle inhibitor XL413 (XL) post-EP (24 h) and N + S pre- (4 h) and post-EP (48 h) under normal culture conditions (37 °C) or cold shock (32 °C for 48 h post-EP). **b** Ratio of HDR/MutEJ repair outcomes measured in **a**. **c** DNA repair outcomes and HDR/MutEJ ratios of homozygous (Homo) GFP iPS cells generated in the 409B2 genetic background. Cells were targeted with Y66H-mutant ssODN M only (ssODN M) and treated and quantified as described in **a**. HDR* indicates the frequency of heterozygous double-positive BFP/GFP. **d** Ratio of HDR/MutEJ repair outcomes measured in **c. e** DNA repair outcomes and HDR/MutEJ ratios of homozygous (Homo) GFP iPS cells that were targeted with a combination of Y66H-mutant ssODN M and T65T silent blocking ssODN B (ssODN M + B), and treated and quantified as described in **a**. Here, HDR* includes heterozygous compound double-positive BFP/pGFP repair outcomes. GFP-positive cells consist of unmodified cells and HDR-mediated monoallelic pGFP/GFP, pGFP/indel (Δ), or biallelic pGFP/pGFP repair outcomes. **f** Ratio of HDR/MutEJ repair outcomes measured in **e**. Data are presented as the mean ± S.D. of three technical replicates for each respective treatment. **g** DNA repair outcome frequencies in 409B2 cells of single clones (*n* = 32 or 31 as indicated), cultured either under normal conditions (−), with synergistic cell-cycle inhibitor XL413 (XL) post-EP (24 h) and NU7441 + SCR7 (N + S) pre- (4 h) and post-EP (48 h) (XL + N + S) or with cold shock (32 °C for 48 h post-EP) and N + S (CS + N + S). HDR outcomes represent clones having undergone template-mediated modification on one or both alleles; MutEJ outcomes include all clones having a mutagenic indel on either allele; and wild-type outcomes correspond to clones having both alleles unmodified. Each targeting is labelled with the target gene and desired missense mutation. **h** HDR/MutEJ ratios quantified in **g**, with the same number of single clones (*n* = 32 or 31 as indicated). Source data are provided as a Source Data file.

Furthermore, cell-cycle synchronization with XL413 and DNA repair modulation with N + S treatment again showed evidence of synergistic gene editing enhancing HDR frequencies (Fig. 6). Remarkably, HDR outcomes reached 83.3% during monoallelic editing of heterozygous GFP iPS cells (Fig. 6a, b), and 96.6% during biallelic editing of homozygous GFP iPS cells when combining XL413 and N + S treatment under cold shock conditions (Fig. 6c, d; Supplementary Fig. 7a, b), including 84.8% biallelic HDR editing outcomes. Moreover, 32.2% of cells became compound heterozygotes when editing homozygous GFP iPS cells with mixed ssODN M and B repair templates (Fig. 6e, f; Supplementary Fig. 7c, d). We ultimately verified HDR frequencies of synergistic gene editing at endogenous loci (Supplementary Fig. 8), using combined XL413 and N + S (XL + N + S) or combined cold shock and N + S (32 °C + N + S) compared to untreated (−) baseline HDR levels (Fig. 6g, h). HDR outcomes included clones with template-mediated repair events on one or both alleles, while MutEJ outcomes included clones with an indel on at least one allele. Overall, synergistic gene editing resulted in several-fold increase in HDR frequencies at all targeted loci, confirming broad applicability of this strategy to targeting the human genome (Fig. 6g). At 5 loci (*KCNH2* N588D/N588K, *APRT* M136T, *HES7* R25W, and *PSMB8* G201V), we obtained from 18 to 23 out of 32 clones with HDR alleles under XL + N + S treatment, representing 56 to 72% total HDR efficiency. Interestingly, cell-cycle arrest with XL413 had a stronger effect on HDR rates than cold shock, when combined with N + S treatment. At the other 5 loci (*KCNE1* D85N, *KCNH2* N45D, *SCN5A* A1428S, and *KCNJ11* T293N/T294M), total editing efficiency was low as shown by the greater proportion of unmodified wild-type clones, suggesting poor gRNA activity. In this case, between 0 and 8 out of 32 HDR clones or 0 to 25% HDR efficiency was achieved. Similarly, HDR/MutEJ ratios were improved with synergistic gene editing, with a stronger effect observed in most cases for XL413 compared to cold shock (Fig. 6h). In summary, these results confirm that synergistic gene editing via cell-cycle synchronization and DNA repair pathway modulation is transferrable between electroporation instruments and to other iPS cell lines, resulting in the efficient clonal generation of homozygous and compound heterozygous mutations at multiple endogenous loci.

## Discussion

**Fig. 7:**
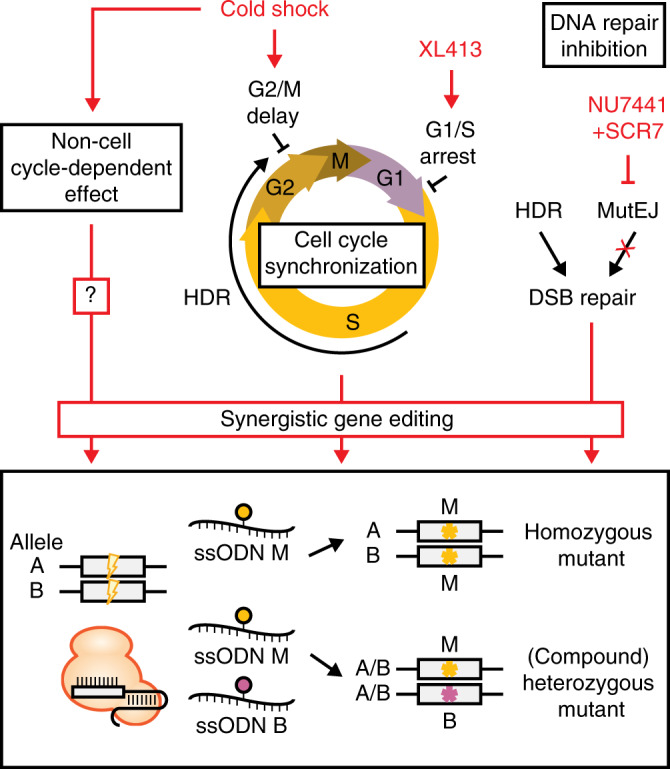
Summary of synergistic gene editing effects favoring HDR outcomes. Synchronization and release of cells into the HDR permissive S and G2 phases of the cell-cycle synergizes with DNA repair inhibition to improve HDR rates. Additional non-cell-cycle-dependent effects of cold shock may also play a role. Leveraging these mechanisms, homozygous mutations are generated at high efficiency, while heterozygous mutations may be generated without indels by using a combination of mutant and blocking ssODN templates.

In this study, we sought to bias DNA repair outcomes towards HDR during ssODN-mediated gene editing in human iPS cells, using chemical and cell-culture condition interventions (Fig. 7). Cold shock was shown to have various effects on cellular function under severe (4–16 °C), moderate (16–25 °C), and mild (25–35 °C) hypothermia, such as slowed metabolism, activation of apoptotic pathways, changes in gene expression and cell-cycle arrest^44,57^. Mild cold shock of 32 °C is characterized by peak levels of cold-inducible RNA-binding protein (CIRP) and RNA-binding motif protein 3 (RBM3) that have important functions in cellular protection against various endogenous and environmental stresses^58–60^. In the context of gene editing, cold shock was shown to improve HDR efficiency^32,33^, yet the mechanism remains unclear. Here we showed that cold shock slowed cell-cycle progression, accumulated cells in G2/M phase, and reduced DNA synthesis rates. Alternatively, cold shock could manifest through other non-cell-cycle-dependent effects such as nuclease stability^30^, gRNA association or DNA cleavage kinetics and stabilization of repair intermediates, post-translational regulation, and cell viability. These additional effects of cold shock may explain the synergy observed in combination with the XL413-induced cell-cycle arrest in G1/S phase.

Regulation of the cell cycle is an ongoing area of interest in the fields of DNA repair and gene editing. It has been proposed that increased CDK activity in S phase stimulates phosphorylation of key HDR proteins such as CtIP, promoting DSB end resection and HDR^61^. Cell-cycle synchronization at the G1/S boundary with Aphidicolin and in late G2/M phase with Nocodazole was shown to enhance HDR frequency^34,35^. Furthermore, simultaneous promotion of G1/S transition by Cyclin D1 (CCND1) overexpression and cell-cycle synchronization in G2/M phase with nocodazole was reported to further improve HDR efficiency^41^. Here, we report enhanced gene editing by the CDC7 inhibitor XL413 in iPS cells. XL413 was reported to accumulate cells in S/G2/M phases in cancer cell lines^46^, while arrest in G1/early S phase was reported in karyotypically normal cells^45^, in agreement with our observations in iPS cells. It is also known that human iPS cells, similar to embryonic stem (ES) cells, have an abbreviated G1 phase of ~2.5 h and enter S phase ~4 h after nocodazole-induced synchronization in G2/M phase^62,63^. During CRISPR-Cas9 targeting, RNP activity is initiated within 4 h of delivery and is mostly completed after ~24 h due to RNP degradation^64^, leaving a limited time window for DSB formation and recruitment of the HDR pathway in S/G2 phase. Therefore, cell-cycle retardation in G2/M or synchronization in G1/early S phase at a time when nuclease activities are high might ensure rapid cell-cycle entry in S phase, which is in any case the predominant cell-cycle state in iPS cells and favors HDR repair. While it is interesting that nocodazole failed to enhance iPS cell gene editing in our experiments, XL413 had a clear positive effect, warranting further exploration.

We found that the NHEJ inhibitors NU7441 and SCR7^41,49^ individually improve HDR efficiency in iPS cells, and further improve HDR efficiency when combined. DNA-PKcs is specifically activated upon DSB formation^61^ and recruits protein components of the NHEJ pathway including DNA ligase IV, which acts to join DNA ends. We suspect that inhibition of DNA-PKcs with NU7441 might circumvent NHEJ complex assembly and allow for alternative DNA repair pathway choices, whereas for downstream inhibition of DNA ligase IV with SCR7, cells may already be committed to NHEJ or some alternative MutEJ repair. This may explain the higher HDR efficiency with NU7441 compared to SCR7 treatment. Interestingly, we did not observe enhancement of HDR by RS-1 or L755507. With regard to the specific type of HDR we are aiming to induce, recent reports speculate that ssODN-mediated DSB repair undergoes synthesis dependent strand annealing (SDSA)^65–67^, which is a RAD51-independent process and thus might explain the limited effect of HDR enhancers RS-1 and possibly L755507 in our system.

Previous studies attempted to combine cell-cycle synchronization with NHEJ inhibition but did not observe improved HDR efficiency compared to individual conditions. This may be due to the use of karyotypically abnormal cell lines as HEK293T cells^40^ or because of the low efficiency of the SCR7 inhibitor alone in pluripotent stem cells^35^. In addition, HDR activity peaks in mid S phase and decreases in G2 phase in karyotypically normal cells^16,17^, which might indicate the limited efficiency we observed in iPS cells with late G2/M phase synchronization by Nocodazole. Taken together, these results demonstrate that different conditions simultaneously modulating cell-cycle phase and DNA repair pathway choice synergistically improve HDR frequency. To our knowledge, we are the first to report synergistic effects of cell-cycle synchronization and DNA repair pathway modulation on precise gene editing.

Using this approach, we obtained up to 96.6% of cells with HDR alleles during biallelic editing, including 84.8% biallelic HDR editing when generating homozygous mutations. Surprisingly, high editing efficiencies were associated with nearly non-existent heterozygous mutant cells. Single HDR alleles formed by precise monoallelic editing were predominantly paired with an indel in the second allele, in agreement with previous reports^11,12^. Therefore, we adopted a mixed ssODN strategy to create compound heterozygous mutant alleles protected from Cas9-recleavage^8,12^, and generated up to 32.2% compound heterozygous mutants at the GFP reporter. Considering endogenous targets for disease modeling, we generated homozygous and compound heterozygous mutations at multiple loci and demonstrated that synergistic gene editing consistently improves HDR outcome frequencies several-fold compared to baseline HDR levels. Eventually, obtaining heterozygous mutants without silent mutations protecting the second allele may require a second round of editing^8,68^, or use of a two-step donor vector targeting and excision process such as the MhAX method^2^. Other strategies could leverage haplotype specificity^69^, conversion tract^8,66^, or nuclease titration to bias towards monoallelic editing, but these approaches will be locus-dependent and come at the cost of lower editing efficiency. We expect that synergistic gene editing will improve ssODN-based strategies for introducing and removing blocking mutations using two-step targeting.

In conclusion, we established a biallelic reporter system capable of resolving allele-specific DNA repair outcomes and defined synergistic gene editing conditions improving HDR and HDR/MutEJ rates using cold shock, cell-cycle synchronization and DNA repair modulation. Leveraging high efficiency biallelic modification, we demonstrated a strategy to generate compound heterozygous iPS cell lines. We expect that improving the reliability of biallelic editing outcomes will greatly facilitate the generation of dominant and recessive genetic disease models and possibly even therapies using human iPS cells.

## Methods

### Human iPS cell culture

Human induced pluripotent stem (iPS) cell lines, namely 409B2 (RIKENBRC #HPS0076) and 1383D6 (RIKEN BRC #HPS1006) were maintained at 37 °C and 5% CO_2_ in commercially available StemFit AK02N medium (Ajinomoto, Cat. No. RCAK02N) on 0.5 mg/mL silk iMatrix-511, Recombinant Human Laminin-511 E8 Fragment (Nippi, Cat. No. 892021) coated tissue culture plates with daily medium exchange. Cell passage was performed every 7 days during maintenance. Cells were first dissociated with Accumax (Innovative Cell Technologies, Cat. No. AM105-500) and 10 min incubation at 37 °C, then washed in StemFit AK02N medium supplemented with 10 µM ROCK inhibitor Y-27632 (Wako, Cat. No. 253-00513) and seeded onto iMatrix511-coated plates at a density of 1 × 10^3^ cells/cm^2^ in StemFit AK02N medium with ROCK inhibitor for 48 h after seeding, and then cultured without ROCK inhibitor. All the cell lines were routinely tested as negative for mycoplasma contamination.

### AAVS1 targeting

Cell lines targeted heterozygously or homozygously to the AAVS1 safe-harbor locus with a CAG-driven GFP cassette were established in two distinct human iPS cell genetic backgrounds, 409B2 and 1383D6. GFP heterozygous (317-A4) and homozygous (317-D6) clones generated in the 409B2 parental iPS cell line were previously reported^43^. GFP heterozygous (073-1-3) and homozygous (073-1-2) clones were generated in the 1383D6 parental iPS cell line as previously described^43^, employing the Neomycin (E182) donor vector and TALE nucleases. Genotyping was performed in targeted clones with Southern blotting using *Sph*I digestion of genomic DNA, internal transgenic or genomic DIG-labelled probes (GFP or 5′ probe), and chemiluminescent detection for 2.5 h.

### iPS cell gene editing

An equimolar amount of crRNA and tracrRNA sequences (IDT, Alt-R CRISPR-Cas9 crRNA and tracrRNA) was hybridized for 5 min at 95 °C to form functional crRNA:tracrRNA duplexes (gRNA). For each electroporation (EP), 30.5 pmol gRNA was mixed with 30.5 pmol Cas9 nuclease (IDT, Alt-R S.p. Cas9 Nuclease V3) (1:1 gRNA:Cas9 ratio) to form RNP complexes, and incubated for 30 min at room temperature (RT). Immediately before EP, 82 pmol ssODN repair templates (IDT, Ultramer DNA Oligonucleotides) was added to the preformed RNP complexes, or equal amounts of mixed ssODN repair templates totaling 82 pmol were added to the RNP when generating compound heterozygous mutants. gRNA and ssODN sequences are listed in Supplementary Tables 1 and 2, respectively. Cells were harvested with Accumax, washed, counted and resuspended at a density of 5 × 10^5^ cells/10 µL in Opti-MEM I reduced-serum medium (Life Technologies, Cat. No. 31985-062). 5 × 10^5^ cells were added to the RNP and ssODN mixture and a total volume of 25 µl was electroporated in a Nepa Electroporation Cuvette 1 mm gap (Nepa Gene, Cat. No. EC-001) using the NEPA21 Electroporator (Nepa Gene) instrument (Poring pulse: 125 V voltage, 2.5 ms pulse length, 50 ms pulse gap, 2 pulses, 10% pulse decay, +orientation; Transfer pulse: 20 V voltage, 50 ms pulse length, 50 ms pulse gap, 5 pulses, 40% pulse decay, ±orientation). Electroporated cells were then transferred to an iMatrix511-coated plate in StemFit AK02N medium supplemented with ROCK inhibitor and incubated at 37 °C, or at 32 °C for 48 h for cold shock treatment and then incubated at 37 °C. Medium exchange was performed after 48 h with StemFit AK02N without ROCK inhibitor, and cells were maintained normally until FACS analysis on day 8. When editing endogenous loci in 409B2 iPS cells, RNP complexes were formed using 61 pmol gRNA and 61 pmol Cas9 (maintaining 1:1 gRNA:Cas9 ratio), an equal amount of 82 pmol ssODN repair templates was added to the RNP before EP, or alternatively 41 pmol of either mixed ssODN repair templates when generating compound heterozygous mutants. Then, 1 × 10^6^ cells were added to the RNP and ssODN mixture and a total volume of 40 µl was electroporated in a Nepa Electroporation Cuvette 1 mm gap on the NEPA21 Electroporator under the same electroporation conditions as outlined above.

In the case of plasmid-based delivery, a target-specific gRNA sequence (GFPx199 gRNA: caccGCTGAAGCACTGCACGCCGT sense and aaacACGGCGTGCAGTGCTTCAGC anti-sense oligos) was cloned into the pX459v2 vector (a gift from F. Zhang, Addgene #62988)^70^. Then, 3 µg of targeting vector and 3 µg (98 pmol) of ssODN repair templates were mixed, 1 × 10^6^ cells resuspended in Opti-MEM were added to the mixture and a total volume of 100 µl was electroporated in a Nepa Electroporation Cuvette 2 mm gap (Nepa Gene, Cat. No. EC-002) using the NEPA21 Electroporator (Poring pulse: 125 V voltage, 5 ms pulse length, 50 ms pulse gap, 2 pulses, 10% pulse decay, +orientation; Transfer pulse: 20 V voltage, 50 ms pulse length, 50 ms pulse gap, 5 pulses, 40% pulse decay, ±orientation). Electroporated cells were then transferred to an iMatrix511-coated plate in StemFit AK02N medium supplemented with ROCK inhibitor and treated with 1 µg/mL puromycin (Merck, Cat. No. P7255-25MG) 24 h post-EP for 48 h. Medium exchange was then performed using StemFit AK02N without ROCK inhibitor, and cells were standardly maintained until FACS analysis on day 8.

For gene editing experiments performed on the MaxCyte STX (MaxCyte) instrument, cells were first harvested 1 day before EP and plated at a density of 1.5 × 10^6^ cells on iMatrix511-coated 6-well plates in StemFit AK02N medium supplemented with ROCK inhibitor, as recommended by the manufacturer′s instructions. On the day of electroporation, RNP complexes were formed in individual 1.5 mL tubes by mixing 61 pmol gRNA with 61 pmol Cas9 nuclease (1:1 gRNA:Cas9 ratio), and 328 pmol ssODN repair templates. Cells were harvested, counted and resuspended in MaxCyte buffer (MaxCyte, Cat. No. EPB-1) at a density of 2 × 10^6^ cells/100 µl, then 1 × 10^6^ cells were added to the RNP and ssODN mixture and a total volume of 50 µl was electroporated in an OC-100 (MaxCyte, Cat. No. SOC-1) or OC-100 × 2 (MaxCyte, Cat. No. SOC-1 × 2) processing assembly on the MaxCyte STX (Optimized protocol 8). After electroporation, processing assemblies were incubated for 20 min at 37 °C, then electroporated cells were transferred to an iMatrix511-coated plate in StemFit AK02N medium supplemented with ROCK inhibitor and incubated at 37 °C, or at 32 °C for cold shock treatment. Medium exchange was performed after 48 h with StemFit AK02N without ROCK inhibitor, and cells were standardly maintained until FACS analysis on day 8.

### Small molecule treatment

For screening and optimization purposes, 5 µg Cas9 (30.5 pmol) was set as a standard editing condition, although higher Cas9 amounts resulted in higher editing efficiency. All small molecules were dissolved in dimethylsulfoxide (DMSO) as recommended by the manufacturer′s instructions, except for XL413 that was dissolved in water. Small molecules were supplemented in StemFit AK02N medium at a final concentration of 3 µM for KU-55933 (Selleck Chemicals, Cat. No. S1092), 1 µM for VE-821 (Selleck Chemicals, Cat. No. S8007), 2 µM for NU7441 (Tocris Bioscience, Cat. No. 3712), 3 µM for Mirin (Sigma–Aldrich, Cat. No. M9948-5MG), 10 µM for PFM01 (Sigma–Aldrich, Cat. No. SML1735-5MG), 20 µM for TDRL-505 (Calbiochem, Cat. No. 530535), 10 µM for RS-1 (Sigma–Aldrich, Cat. No. R9782-5MG), 1 µM for SCR7 (Xcess Bioscience, Cat. No. M60082-2S) and 5 µM for L755507 (Sigma–Aldrich, Cat. No. SML1362-5MG), which are the maximum non-toxic concentrations in iPS cells defined by a titration curve and colony count after 48 h treatment. XL413 (Sigma–Aldrich, Cat. No. SML1401-5MG) was supplemented at a final concentration of 33 µM, and Nocodazole (Sigma–Aldrich, Cat. No. M1404-2MG) at 100 ng/mL. When combining several small molecules, the concentrations were equivalent to individual treatments. The DMSO control was supplemented at a volume equal to the largest volume of individual or combined small molecules used in treated conditions. For gene editing, cells were pretreated for 4 h before EP and for 48 h after EP (pre) with each small molecule, or when specified only for 48 h after EP without pretreatment (post). Pretreated cells were harvested and resuspended in medium supplemented with small molecule during cell counting, before being resuspended in Opti-MEM medium for electroporation. After EP, cells were transferred to an iMatrix511-coated plate in StemFit AK02N medium supplemented with ROCK inhibitor and small molecule and incubated at 37 °C, or at 32 °C for 48 h for cold shock treatment and then incubated at 37 °C. Medium exchange was performed 48 h after EP with StemFit AK02N medium, and cells were standardly maintained until FACS analysis on day 8. For cell-cycle synchronization and gene editing, XL413 treatment was performed for 24 h after EP, replaced by StemFit AK02N medium supplemented with ROCK inhibitor for 24 h and then by StemFit AK02N medium without ROCK inhibitor. Nocodazole treatment was applied 16 h before EP, supplemented during cell harvest and counting, and replaced by StemFit AK02N medium supplemented with ROCK inhibitor after EP.

### Cell-cycle analysis

Using the Click-iT EdU Alexa Fluor 647 Flow Cytometry Assay Kit (Thermo Fischer Scientific, Cat. No. C10424), cells were first incubated for 2 h 15 min with 10 µM 5-ethynyl-2′-deoxyuridine (EdU) in addition to the small molecule or culture condition being tested. Cells were then harvested, counted, and an equal number of cells was transferred to a new 1.5 mL tube. According to the manufacturer′s instructions, cells were washed with 1% BSA in 1X DPBS, fixed with Click-iT fixative (4% paraformaldehyde in DPBS), incubated for 15 min at room temperature (RT) and protected from light, and washed with 1% BSA in DPBS. Cells were then permeabilized in 1X Click-iT saponin-based permeabilization and wash reagent. The Click-iT labelling reaction was performed by adding a mixture of Alexa Fluor 647 azide fluorescent dye, 2 mM CuSO_4_ and 1X Click-iT EdU buffer additive to the samples, incubating the reaction mixture for 30 min at RT and protected from light, and then washing and resuspending the samples in 1X Click-iT saponin-based permeabilization and wash reagent. Finally, a propidium iodide (PI) (Wako, Cat. No. 169-26281) staining solution composed of Triton-X 100 (Nacalai Tesque, Cat. No. 282-29) at a final concentration of 0.1%, RNase A at 0.2 mg/mL (Invitrogen, Cat. No. 12091-021) and PI at 20 µg/mL in DPBS was mixed to the samples and incubated for 30 min at RT, protected from light.

### Flow cytometry and cell sorting

Measurement of fluorescence intensities was performed using 5 × 10^5^ cells resuspended in FACS buffer (2% FBS in DPBS) on a BD LSRFortessa Cell Analyzer (BD Biosciences) with BD FACSDiva software (BD Bioscience), and raw data were analyzed with FlowJo (FlowJo LLC). For editing experiments in GFP iPS cells, cells were acquired using the Pacific Blue (450/50 nm) and FITC (530/30 nm) filters. Cells stained with EdU and PI were acquired using Alexa Fluor 647 (660/20 nm) and PE-Tx-Red-GR filters. For characterization of DNA repair outcomes, BFP+, BFP−/GFP−, and GFP+ populations were sorted from edited heterozygous GFP iPS cells, and for the clonal isolation of compound heterozygous cells, the BFP+/GFP+ population was sorted from edited homozygous GFP iPS cells targeted with mixed ssODN repair templa/cates. Cells were harvested in FACS buffer at a density of 1 × 10^6^ cells/mL and filtered through a cell-strainer for dissociation. Sorting gates were set for singlets, and the desired populations were collected on a BD FACSAria II cell sorter (BD Bioscience) into StemFit AK02N medium containing 10-20 µM ROCK inhibitor.

### HDR quantification assay

Ouabain octahydrate (Sigma–Aldrich, Cat. No. O3125-250MG) was dissolved in water. Cells targeted at the ATP1A1 locus and being tested for small molecule or culture conditions were treated 48 h after EP with 1 mM Ouabain in StemFit AK02N medium supplemented with ROCK inhibitor until day 6, in order to selectively obtain HDR-edited colonies. Cells were then maintained normally with StemFit AK02N medium until day 13, and forming colonies were stained with crystal violet. Tissue culture plates were placed on ice, washed twice with ice-cold 1X DPBS, fixed with ice-cold methanol for 10 min, then cells were removed from the ice and incubated with 0.05-0.1% Crystal Violet (Nacalai, Cat. No. 09804-52) in 25–100% methanol for 10 min at RT. Excess Crystal Viotel was removed by washing with water, and the plates photographed for manual colony counting.

### Clonal analysis

For characterization of DNA repair outcomes of single clones, cells were harvested on day 7 after EP and 5 × 10^5^ cells were used for genomic DNA extraction (described below), two cryogenic tubes with 5 × 10^5^ cells each resuspended in STEM-CELLBANKER GMP grade (TAKARA BIO, Cat. No. CB047) were frozen down for total population stocks, and 300–600 cells were plated in iMatrix511-coated 6 cm dishes. Ten days after plating, colonies ~1 mm in diameter were picked in 5 µl of media under the microscope and transferred to a 96-well PCR plate. Afterwards, 10 µl of QuickExtract DNA Extraction Solution (Epicenter, Cat. No. QE09050) was added, and plates were incubated for 6 min at 65 °C then 2 min at 98 °C before being stored at −30 °C.

### Genotyping

For genomic DNA extraction, 0.5–1 × 10^6^ cells were washed with 1X DPBS, DNA was purified using the DNeasy Blood & Tissue Kit (Qiagen, Cat. No. 69506) as recommended by manufacturer′s instructions, and purified DNA was resuspended in 100 µl of water. Target sequences were amplified using KAPA HiFi HS ReadyMix (Kapa Biosystems, Cat. No. KK2602) polymerase chain reaction (PCR), enzymatic PCR product cleanup was performed with ExoSAP-IT Express reagent (Thermo Fischer Scientific, Cat. No. 75001), and Sanger sequencing was performed using the BigDye Terminator v3.1 CS Kit (Thermo Fischer Scientific, Cat. No. 4337456) according to the manufacturer′s instructions. Genotyping primers are listed in Supplementary Table 3. Reactions were then purified by ethanol precipitation and acquired on a 3130*xl* Genetic Analyzer (Applied Biosystems). Sequence alignments were analyzed with Snapgene (GSL Biotech LLC), and sequence trace files with low base calling confidence were excluded from analyses.

### RFLP analyses

ssODN-mediated modification of the GFP target sequence was verified by Restriction Fragment Length Polymorphism (RFLP) of a newly created silent restriction site. The target sequences were PCR amplified from purified gDNA using KAPA HiFi HS ReadyMix, then amplicons were cleaved with FastDigest *Nco*I (Thermo Fischer Scientific, Cat. No. FD0573) restriction enzyme in FastDigest Buffer (Thermo Fischer Scientific, Cat. No. B64) and incubation for 30 min at 37 °C. Cleaved amplicons were then resolved by gel electrophoresis, acquired on an AE-9000N E-Graph (Atto) gel documentation system and quantified with the ImageJ software.

### TIDE analysis

TIDE analysis was performed on mixed sequences using the online tool at https://tide.nki.nl/^71^. Sequence data from 409B2-derived heterozygous GFP iPS cells were used as a reference. The deletion size window was extended to 50 bp to visualize larger deletions, while other parameters were kept as default.

### Fluorescent microscopy

Cells plated on iMatrix511-coated 6-well plates with StemFit AK02N medium were directly imaged using an LSM 710 (ZEISS) confocal microscope and LSM Software ZEN 2009 (ZEISS). Images were obtained with a ×10 objective, and GFP and DAPI filters with appropriate gain and exposure times.

### Reporting summary

Further information on research design is available in the Nature Research Reporting Summary linked to this article.

## Supplementary information

Supplementary Information

Reporting Summary

Peer Review File

## Data Availability

The data supporting the findings of this study are available from the corresponding author upon request. The source data underlying Figs. 1–6 and Supplementary Figs. 3–7 are provided as a Source Data file. The publicly available web tool TIDE (https://tide.nki.nl/) was used in this study.

## Source data

Source Data
