# Supplementary material for: Synergistic gene editing in human iPS cells via cell cycle and DNA repair modulation
Source: Nat Commun. 2020 Jun 8;11:2876. doi: 10.1038/s41467-020-16643-5 (PMC7280248; doi:10.1038/s41467-020-16643-5)
Supplement: Supplementary file 1 — Supplementary Information [file 41467_2020_16643_MOESM1_ESM.pdf]

SUPPLEMENTARY INFORMATION

Synergistic gene editing in human iPS cells via cell cycle and DNA repair modulation

Maurissen et al.

This file includes:

Supplementary Figures 1-8
Supplementary Tables 1-3

SUPPLEMENTARY FIGURES

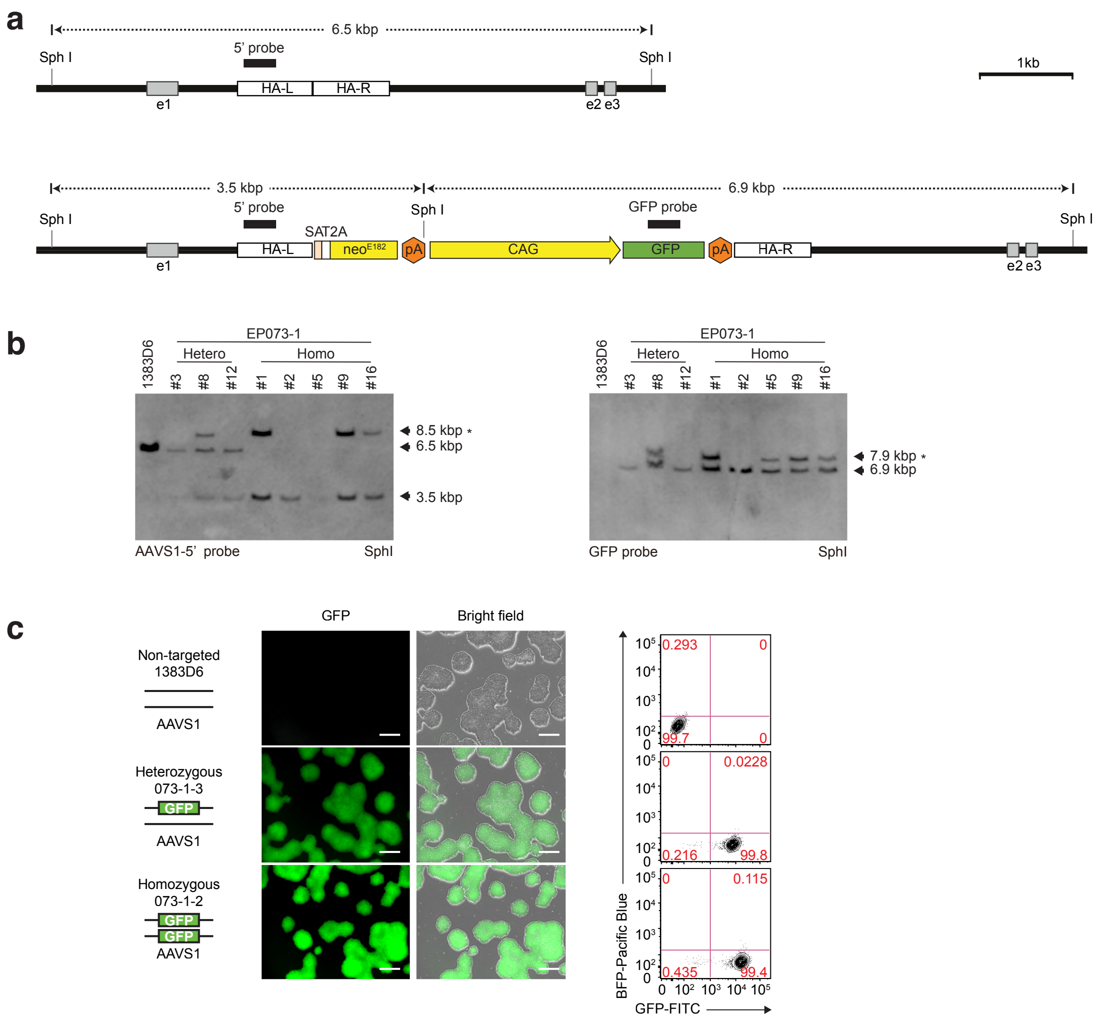

Supplementary Fig. 1: Generation of GFP targeted clones. a Schematic of the AAVS1 locus before (top) and after targeting with a CAG-driven GFP cassette (bottom). The expected fragment sizes from *Sph*I digestion, as well as the positions of genomic or internal transgenic Southern blot probes (5' or GFP probe) are marked. Gray boxes correspond to *PPP1R12C* exons. HA-L/R, left/right homology arms; SA, splice acceptor; T2A, *Thosea asigna* virus 2A peptide; pA, bovine growth hormone or rabbit b-globin polyadenylation signals. **b** Southern blot analysis of *Sph*I-digested genomic DNA with a 5' genomic probe (left) or an internal GFP probe (right). Targeted alleles were detected by the presence of a 3.5kbp (5' probe) or a 6.9kbp (GFP probe) product, while normal alleles conserved a 6.5kbp product detected with the 5' probe. Clones with aberrant banding due to donor backbone integration predicted and observed at 8.5kbp (5' probe) and 7.9kbp (GFP probe) are indicated with an asterisk (*). The heterozygous 073-1-3 (#3) and homozygous 073-1-2 (#2) clones were selected for further gene editing experiments. Southern blots are from one membrane and represent one incremental exposure. **c** Schematic of the AAVS1 genotype (left), fluorescence microscopy images with brightfield (middle) and GFP fluorescence intensity by FACS (right) of the non-targeted 1383D6 parent iPS cell line (top), heterozygous (073-1-3; center) and homozygous

(073-1-2; bottom) cell lines targeted with GFP. Scalebars are 500µm. Representative images of more than 3 independent experiments with similar results are shown.

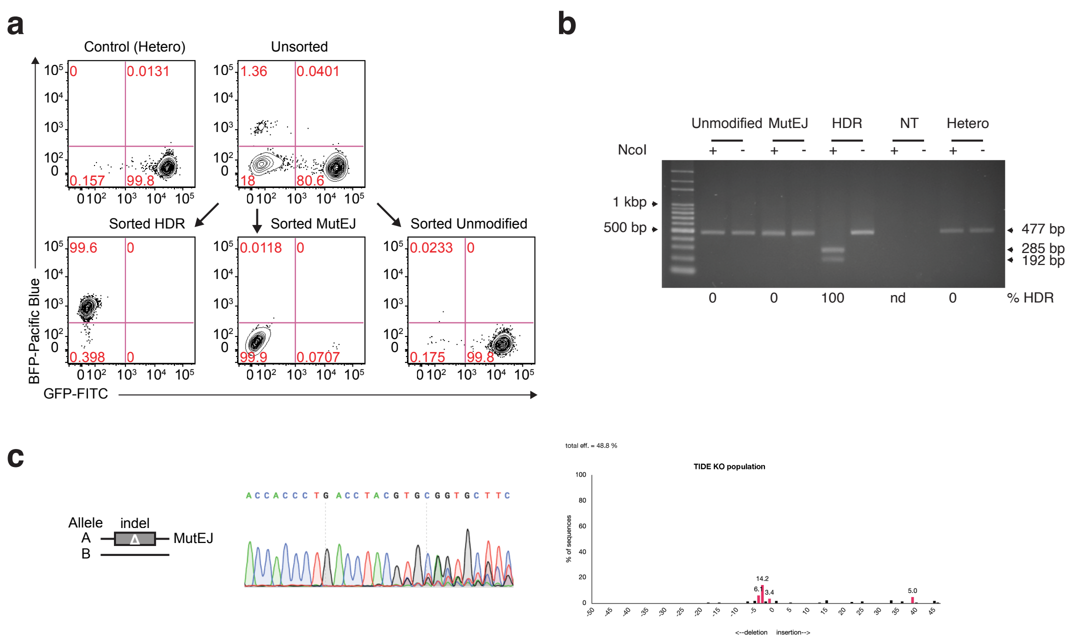

Supplementary Fig. 2: Characterization of the fluorescent DNA repair assay. a Targeting of heterozygous GFP iPS cells with plasmid expressing Cas9 and GFPx199 and with ssODN (top middle), selection for 48h with Puromycin, and FACS sorting of HDR (BFP+; bottom left), MutEJ (double negative; bottom middle) and unmodified (GFP+; bottom right) DNA repair outcomes. FACS of lower panels was performed 1 week after sorting on expanded cells. The control shows untargeted heterozygous GFP iPS cells (top left). **b** Restriction Fragment Length Polymorphism (RFLP) assay of a *Nco*I restriction site introduced by ssODN-mediated targeting, for non-targeted (NT) control, heterozygous GFP (Hetero) control and sorted HDR, MutEJ and unmodified outcomes obtained in **a**. Cleavage product quantification shows the percentage of HDR outcomes. RFLP analysis was performed once. **c** Scheme (left) and sequence (middle) of sorted heterozygous MutEJ cells, and TIDE analysis showing the resulting indel pattern (right).

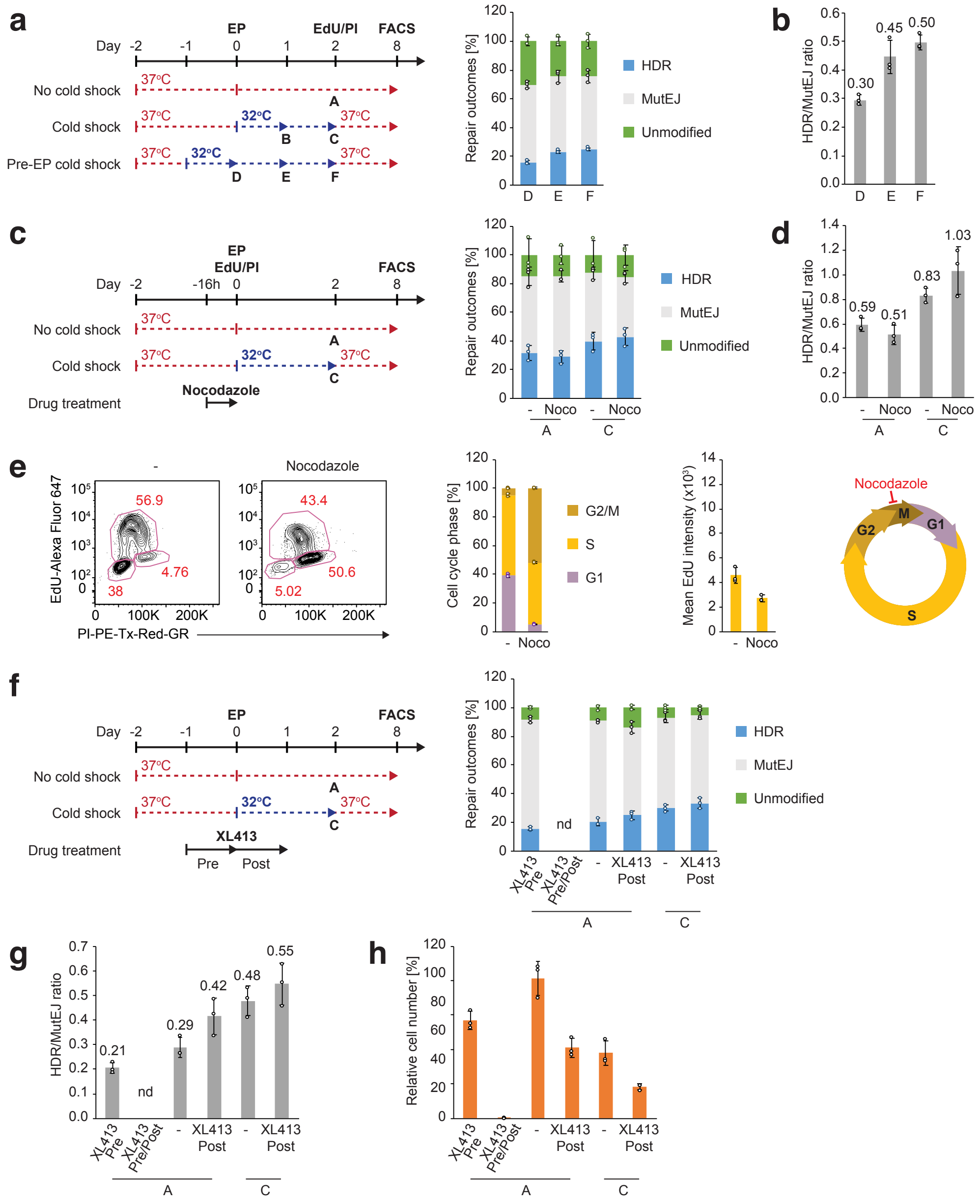

Supplementary Fig. 3: Effect of cell cycle synchronization on HDR efficiency. a Experimental timeline with no cold shock [A], and cold shock for 24h [B] or 48h [C] following electroporation (EP), or cold shock for 24h before EP only [D], or for 24h [E] or 48h [F] following EP (left). The resulting effect on DNA repair outcome frequency is shown for conditions [D-F] (right). **b** Ratio of HDR/MutEJ repair outcomes measured in **a**. **c** Cell cycle synchronization in G2/M with Nocodazole does not affect HDR efficiency in iPS cells. Experimental timeline of Nocodazole treatment for 16h pre-EP, under normal culture [A] or cold shock [C] condition following EP on day 0 (left). EdU/PI staining was performed before EP, and FACS analysis on day 8. The resulting effect on DNA repair outcome frequency is shown (right). **d** Ratio of HDR/MutEJ repair outcomes measured in **c**. **e** Representative FACS plots of EdU/PI staining (left) before EP for untreated and Nocodazole-treated cells, and quantification of cell cycle phase (middle left) and mean EdU intensity of S-phase cells (middle right). Schematic of Nocodazole-induced cell cycle arrest in the late G2/M phase (right). **f** Cell cycle synchronization at the G1/S

boundary with XL413 improves HDR efficiency in iPS cells. Experimental timeline of XL413 treatment for 24h pre- (Pre) or post-EP (Post) following EP, under normal culture [A] or cold shock [C] conditions (left). The resulting effect on DNA repair outcome frequency is shown (right). **g** Ratio of HDR/MutEJ repair outcomes measured in **f**. **h** Relative cell number normalized to untreated control, obtained from **f**. XL413 Pre/Post treatment resulted in cell death and non-detectable (nd) repair outcomes in **f** and **g**. All data are presented as the mean ± S.D. of three technical replicates for each respective treatment. Source data are provided as a Source Data file.

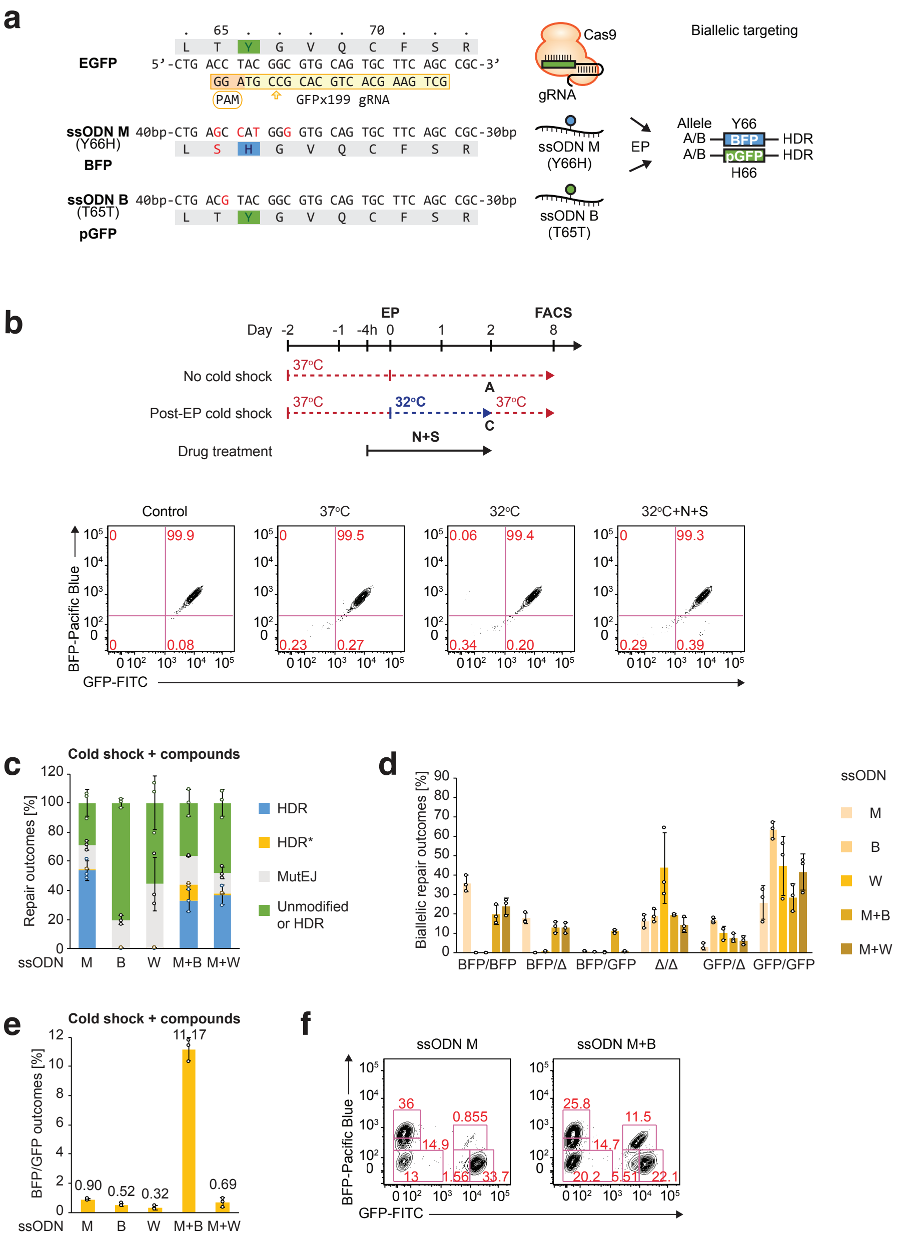

Supplementary Fig. 4: Heterozygous compound mutations prevent Cas9 re-cleavage. a Generation of a BFP/pGFP double-positive clone using Y66H mutant ssODN M and T65T silent blocking ssODN B in homozygous GFP iPS cells. Double-positive BFP/GFP cells were sorted on day 8 after targeting, and clones were picked and analyzed. **b** Experimental timeline of a BFP/pGFP clone targeted with GFPx199 gRNA to measure Cas9-mediated re-cleavage activity of edited BFP and protected-GFP alleles under normal culture, and cold shock conditions, and combined NU7441+SCR7 (N+S) treatment (top). FACS plots of an untargeted BFP/pGFP clone (Control) and re-targeting under normal culture [A], cold shock [C], and cold shock combined with N+S treatment (32°C +N+S) during FACS analysis on day 8 (bottom). **c** GFP editing with ssODN M, B or W individually, or in M+B and M+W combination, and effect on DNA repair outcome frequency under combined cold shock and N+S treatment. HDR* indicates the frequency of heterozygous double-positive BFP/GFP or compound heterozygous double-

positive BFP/pGFP repair outcomes. GFP-positive cells include unmodified cells and HDR-mediated monoallelic pGFP/GFP, pGFP/indel (Δ) or biallelic pGFP/pGFP repair outcomes. **d** Distribution of biallelic repair outcomes shown in **c**. **e** Biallelic BFP/GFP repair outcome frequency measured in **d**. **f** Representative FACS plots of BFP conversion only (ssODN M) or heterozygous compound BFP/pGFP mutations (ssODN M+B). Data are presented as the mean ± S.D. of three technical replicates of independent electroporations for each respective condition. Source data are provided as a Source Data file.

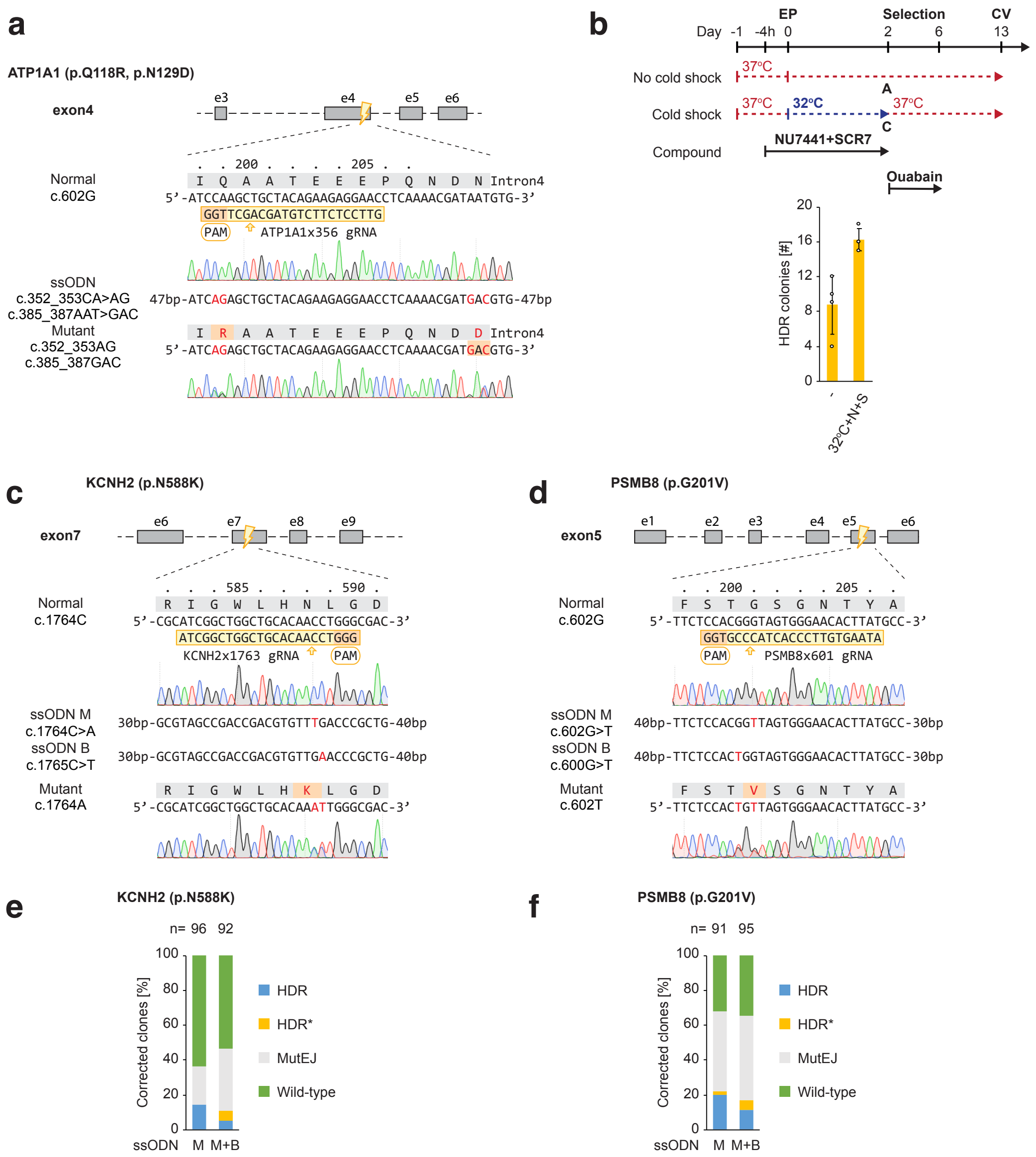

Supplementary Fig. 5: Generation of heterozygous mutations at endogenous loci. a Target sequence and editing strategy at the *ATP1A1* locus to generate p.Q118R and p.N129D mutations. Shown are Sanger sequences of the *ATP1A1* locus before and after HDR editing. **b** Experimental timeline for *ATP1A1* editing including cold shock, compound treatment and ouabain selection (left). Quantification of HDR colony formation with combined cold shock [C] and N+S drug treatment (right). Data are presented as the mean ± S.D. of four technical replicates for each respective treatment. **c** Target sequence at the *KCNH2* locus and strategy to recreate a heterozygous p.N588K pathogenic mutation with an ssODN encoding a missense mutation (ssODN M) or a silent block (ssODN B) using combination of cold shock and N+S treatment. Shown are Sanger sequences of the *KCNH2* locus before and after HDR editing with mixed ssODN repair templates. **d** Target sequence at the *PSMB8* locus and strategy to recreate a heterozygous p.G201V pathogenic mutation. Likewise, shown are Sanger sequences of the *PSMB8* locus before and after HDR editing with mixed ssODN repair templates. **e** Quantification of the editing efficiency of compound heterozygous, homozygous

and heterozygous mutations obtained from clonal analysis of editing with ssODN M (n=96) or ssODN M+B (n=92). **f** ssODN design and quantification is similar to **e** with ssODN M (n=91) or ssODN M+B (n=95). Source data are provided as a Source Data file.

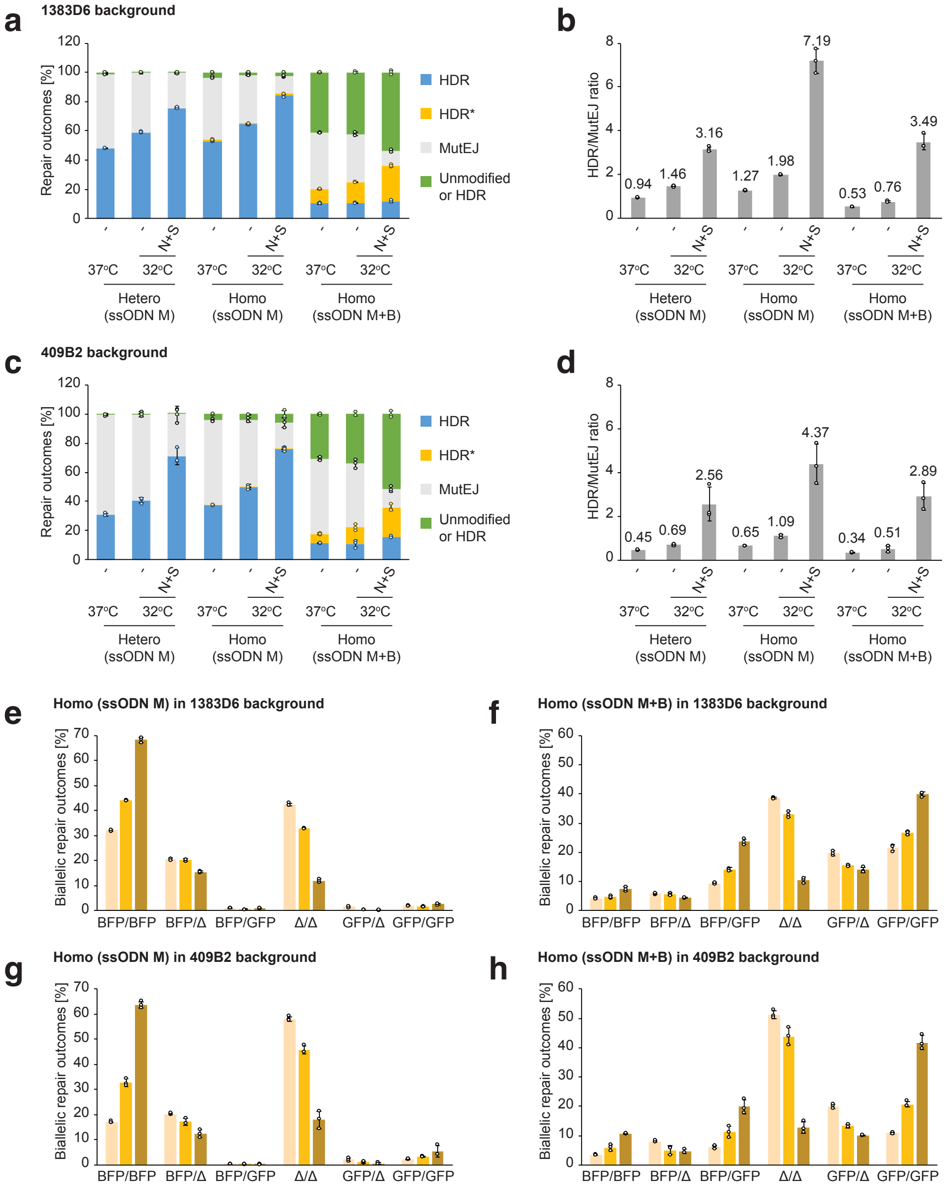

Supplementary Fig. 6: Increased editing efficiency with MaxCyte electroporation. a DNA repair outcome frequency using the MaxCyte electroporation instrument. Heterozygous (Hetero) and homozygous (Homo) GFP iPS cells generated in the 1383D6 genetic background were targeted with Y66H mutant ssODN M only (ssODN M), and homozygous GFP iPS cells were targeted with a combination of Y66H mutant ssODN M and T65T silent blocking ssODN B (ssODN M+B) under normal culture (37°C) or cold shock (32°C for 48h post-EP) conditions, or cold shock combined with N+S treatment. HDR* indicates the frequency of heterozygous double-positive BFP/GFP and heterozygous compound double-positive BFP/pGFP repair outcomes. In the case of homozygous GFP iPS cells targeted with ssODN M+B, GFP-positive cells include unmodified cells and HDR-mediated monoallelic pGFP/GFP, pGFP/indel (Δ) or biallelic pGFP/pGFP repair outcomes. **b** Ratio of HDR/MutEJ repair outcomes measured in **a**. **c**-**d** Same than **a**-**b** with heterozygous and homozygous GFP iPS cells generated in the 409B2 genetic background. **e** Biallelic repair outcome frequencies obtained from **a** in homozygous

GFP iPS cells targeted with ssODN M in the 1383D6 genetic background under normal culture or cold shock conditions, or cold shock combined with N+S treatment. **f** Same as in **e** in homozygous GFP iPS cells targeted with ssODN M+B in the 1383D6 genetic background, and effect on biallelic BFP/BFP and compound heterozygous BFP/pGFP efficiencies under normal culture or cold shock conditions, or cold shock combined with N+S treatment. **g**-**h** Same than **e**-**f** with homozygous GFP iPS cells generated in the 409B2 genetic background. All data are presented as the mean ± S.D. of three technical replicates for each respective treatment. Source data are provided as a Source Data file.

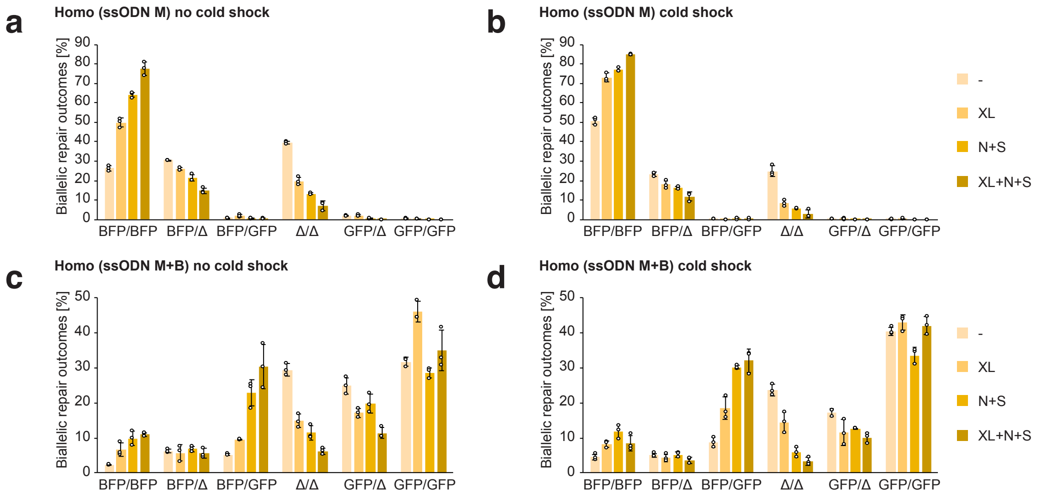

Supplementary Fig. 7: Synergistic gene editing with MaxCyte electroporation. a Biallelic repair outcome frequencies obtained from homozygous GFP iPS cells targeted with ssODN M during single or combination treatment with cell cycle inhibitor XL413 (XL) and N+S under normal culture conditions. **b** Same than in **a** but under cold shock conditions. **c** Biallelic repair outcome frequencies obtained from homozygous GFP iPS cells targeted with ssODN M+B under normal culture conditions. **d** Same than in **c** but under cold shock conditions. All data are presented as the mean ± S.D. of three technical replicates for each respective treatment. Source data are provided as a Source Data file.

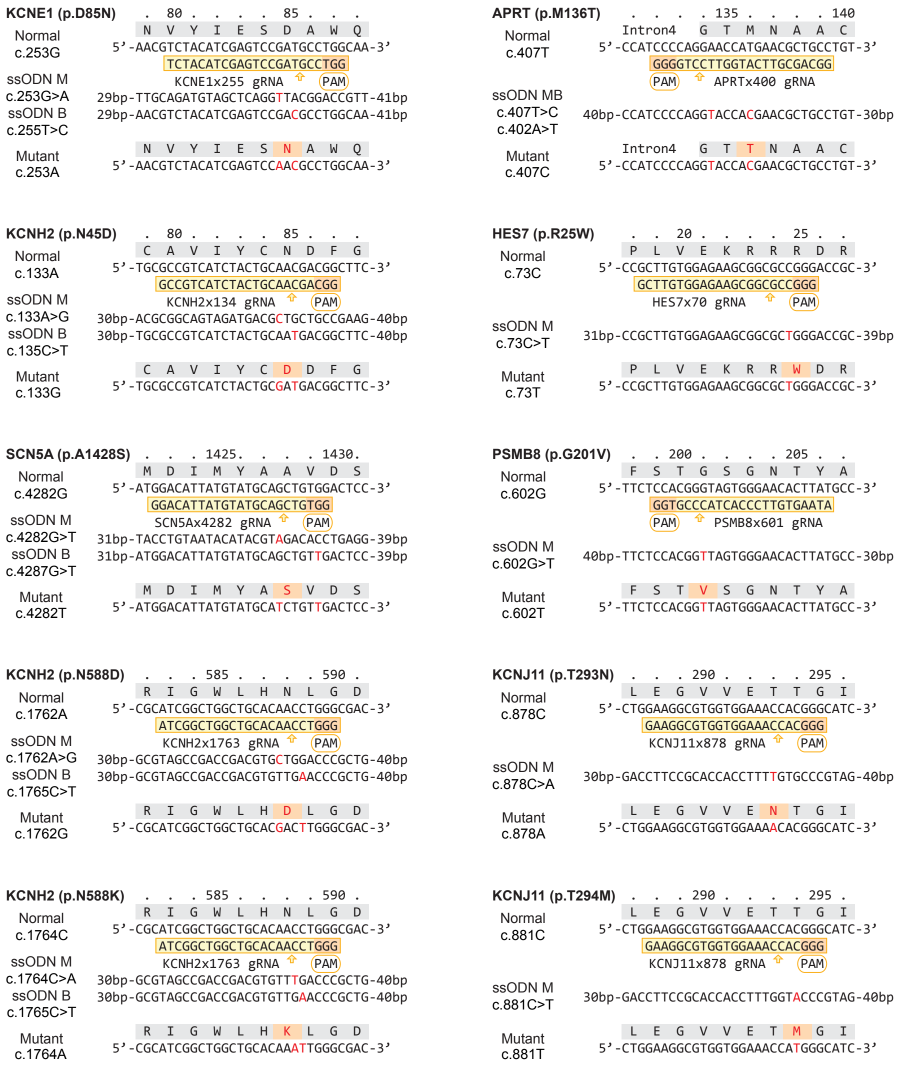

Supplementary Fig. 8: Sequence designs of endogenous loci targeted with synergistic gene editing and MaxCyte electroporation. Target sequence and editing strategy for all 10 targeting experiments including gRNA and ssODN template sequences, as well as the missense mutations being generated.

SUPPLEMENTARY TABLES

Supplementary Table 1: gRNA.

Gene	Target	Name	Sequence
EGFP	c.196T>C	GFPx199	GCTGAAGCACTGCACGCCGT
ATP1A1	RD	ATP1A1x356	GTTCCTCTTCTGTAGCAGCT
KCNH2	c.1764C>A	KCNH2x1763	ATCGGCTGGCTGCACAACCT
PSMB8	c.602G>T	PSMB8x601	ATAAGTGTTCCCACTACCCG
KCNE1	c.253G>A	KCNE1x255	TCTACATCGAGTCCGATGCC
KCNH2	c.133A>G	KCNH2x134	GCCGTCATCTACTGCAACGA
SCN5A	c.4282G>T	SCN5Ax4282	GGACATTATGTATGCAGCTG
KCNH2	c.1762A>G	KCNH2x1763	ATCGGCTGGCTGCACAACCT
APRT	c.407T>C	APRTx400	GGCAGCGTTCATGGTTCCTG
HES7	c.73C>T	HES7x70	GCTTGTGGAGAAGCGGCGCC
KCNJ11	c.878C>A	KCNJ11x878	GAAGGCGTGGTGGAAACCAC
KCNJ11	c.881C>T	KCNJ11x878	GAAGGCGTGGTGGAAACCAC

Supplementary Table 2: ssODN templates.

Gene	Target Site	Name	Sequence
EGFP	c.196T>C	GFPc.196C-50/50-t	CGGCAAGCTGCCCGTGCCCTGGCCCACCCTCGTG<br>ACCACCCTGAGCCATGGGGTGCAGTGCTTCAGCC<br>GCTACCCCGACCACATGAAGCAGCACGACTTC
	c.195C>G	GFP-50/50-b-t	CGGCAAGCTGCCCGTGCCCTGGCCCACCCTCGTG<br>ACCACCCTGACGTACGGCGTGCAGTGCTTCAGCC<br>GCTACCCCGACCACATGAAGCAGCACGACTTC
	c.196T	GFP-50/50-t	CGGCAAGCTGCCCGTGCCCTGGCCCACCCTCGTG<br>ACCACCCTGACCTACGGCGTGCAGTGCTTCAGCC<br>GCTACCCCGACCACATGAAGCAGCACGACTTC
ATP1A1	RD	ATP1A1-RD	CAATGTTACTGTGGATTGGAGCGATTCTTTGTTTC<br>TTGGCTTATAGCATCAGAGCTGCTACAGAAGAG<br>GAACCTCAAAACGATGACGTGAGTTCTGTAATTC<br>AGCATATCGATTTGTAGTACACATCAGATATCTT
KCNH2	c.1764C>A	KCNH2-N588K-50/50-t	GGCCGCCCAGGCCGCTGCTGTTGTAGGGTTTGCC<br>TATCTGGTCGCCCAGTTTGTGCAGCCAGCCGATG<br>CGTGAGTCCATGTGTGGCTGCTCCATGTTGCC
	c.1765C>T	KCNH2-L589L-50/50-t	GGCCGCCCAGGCCGCTGCTGTTGTAGGGTTTGCC<br>TATCTGGTCGCCCAAGTTGTGCAGCCAGCCGATG<br>CGTGAGTCCATGTGTGGCTGCTCCATGTTGCC
PSMB8	c.602G>T	PSMB8-G201V-50/50-t	CTACGTGGATGAACATGGGACTCGGCTCTCAGG<br>AAATATGTTCTCCACGGTTAGTGGGAACACTTAT<br>GCCTACGGGGTCATGGACAGTGGCTATCGGCCT

	c.600G>T	PSMB8-T200T-50/50-t	CTACGTGGATGAACATGGGACTCGGCTCTCAGG AAATATGTTCTCCACTGGTAGTGGGAACACTTAT GCCTACGGGGTCATGGACAGTGGCTATCGGCCT
KCNE1	c.253G>A	KCNE1-D85N-50/50-t	CTCTCCAGGACCCGGGCCTGGACATAGGCCTTGT CCTTCTCTTGCCAGGCATTGGACTCGATGTAGAC GTTGAATGGGTCGTTCGAGTGCTCCAGCTTCT
	c.255T>C	KCNE1-D85D-50/50-nt	AGAAGCTGGAGCACTCGAACGACCCATTCAACG TCTACATCGAGTCCGACGCCTGGCAAGAGAAGG ACAAGGCCTATGTCCAGGCCCGGGTCCTGGAGA G
KCNH2	c.133A>G	KCNH2-N45D-50/50-t	GCTGCATCACCTCGGCCCGCGAGTAGCCGCACA GCTCGCAGAAGCCGTCGTCGCAGTAGATGACGG CGCAGTTCTCCACCCGAGCGTTGGCGATGATGAA
	c.135C>T	KCNH2-N45N-50/50-nt	TTCATCATCGCCAACGCTCGGGTGGAGAACTGCG CCGTCATCTACTGCAATGACGGCTTCTGCGAGCT GTGCGGCTACTCGCGGGCCGAGGTGATGCAGC
SCN5A	c.4282G>T	SCN5A-A1428S-50/50-t	GACTTGGTGGAAGAAGCCACTGTGGCAACCTAC CCCCCTGGAGTCCACAGATGCATACATAATGTCC ATCCAGCCTTTAAATGTTGCCTGGGAGGAAAAG
	c.4287G>T	SCN5A-V1429V-50/50-nt	CTTTTCCTCCCAGGCAACATTTAAAGGCTGGATG GACATTATGTATGCAGCTGTTGACTCCAGGGGG GTAGGTTGCCACAGTGGCTTCTTCCACCAAGTC
KCNH2	c.1762A>G	KCNH2-N588D-50/50-t	GGCCGCCCAGGCCGCTGCTGTTGTAGGGTTTGCC TATCTGGTCGCCCAGGTCGTGCAGCCAGCCGATG CGTGAGTCCATGTGTGGCTGCTCCATGTTGCC
	c.1765C>T	KCNH2-L589L-50/50-t	GGCCGCCCAGGCCGCTGCTGTTGTAGGGTTTGCC TATCTGGTCGCCCAAGTTGTGCAGCCAGCCGATG CGTGAGTCCATGTGTGGCTGCTCCATGTTGCC
APRT	c.407T>C c.402A>T	APRT-M136T-50/50-t	CTGCTCTCTGCAGCCCAGGCCAACTGGGGACCTC ACCCTCCCATCCCCAGGTACCACGAACGCTGCCT GTGAGCTGCTGGGCCGCCTGCAGGCTGAGGTC
HES7	c.73C>T	HES7-R25W-50/50-nt	CTCCTTTTCTCGCTGGTCGCAGATGCTCAAGCCGC TTGTGGAGAAGCGGCGCTGGGACCGCATCAACC GCAGCCTGGAAGAGCTGAGGCTGCTGCTGCTG
KCNJ11	c.878C>A	KCNJ11-T293N-50/50-t	GGATCTCATCGGCCAGGTAGGAGGTGCGGGCCT GGGTGGTGATGCCCGTGTTTTCCACCACGCCTTC CAGGATGACGATGATCTCGAGGTCCTGGTGGTG
KCNJ11	c.881C>T	KCNJ11-T294M-50/50-t	GGATCTCATCGGCCAGGTAGGAGGTGCGGGCCT GGGTGGTGATGCCCATGGTTTCCACCACGCCTTC CAGGATGACGATGATCTCGAGGTCCTGGTGGTG

Supplementary Table 3: Genotyping primers.

Gene	Target Site	Name	Sequence
EGFP	c.196T>C	dna549	AGCAAGGGCGAGGAGCTGTT
		dna649	GCCGTTCTTCTGCTTGTCGG
ATP1A1	RD	dna2778-ATP1A1_Fwd	TATTGCAACCGTCCAGCTAC

		dna2779-ATP1A1_Rev	GAATGGCCACCAAGCATTTC
KCNH2	c.1764C>A	dna2093-HERG-Exon8, 9-F	CTCTGTCCCAAAGCTAGCAC
		dna2094-HERG-Exon8, 9-R	GGGTCCTTACTACTGACTGTGA
PSMB8	c.602G>T	dna3253-PSMB8 p.G201V-R	GGATAAGAAGGTGGGTGCTCT
		dna3252-PSMB8 p.G201V-F	AAACCATATGACTGGGCCTTTA
KCNE1	c.253G>A	dna463-kcne1.3F3	AAACCAAAATGCACACATGCAACC
		dna508-hKCNE1.3R_4696-4669	TGGAAATGTCATGCCTTTAGGTTCAGTC
		dna005-kcne1F3	GTTCAGCAGGGTGGCAACAT
KCNH2	c.133A>G	dna2081-HERG-Exon2-F	CTGTGTGAGTGGAGAATGTGG
		dna2082-HERG-Exon2-R	GTCACACCCCCACAGAAC
SCN5A	c.4282G>T	dna3177-SCN5A-RP	CCTTCTTGGGGCATCTTCTC
		dna3176-SCN5A-FP	CCAGAGGTGGGTAGGGATAG
KCNH2	c.1762A>G	dna2093-HERG-Exon8, 9-F	CTCTGTCCCAAAGCTAGCAC
		dna2094-HERG-Exon8, 9-R	GGGTCCTTACTACTGACTGTGA
APRT	c.407T>C	dna1711-hAPRT-T7F5	GTCGTGGATGATCTGCTGG
		dna1712-hAPRT-T7R5	TGCCCAAGGCTGATATTTCCC
HES7	c.73C>T	dna2227-HES7-Exon2+3-F	GCGAGCTACAGAAACTGATCT
		dna2228-HES7-Exon2+3-R	GGGAGAAAATGAGGGAGACAC
KCNJ11	c.878C>A	dna3116-KCNJ11-FP	GTGGTACGCAAGACCACCAG
		dna3117-KCNJ11-RP	GGGCTACATACCACATGGTCC
KCNJ11	c.881C>T	dna3116-KCNJ11-FP	GTGGTACGCAAGACCACCAG
		dna3117-KCNJ11-RP	GGGCTACATACCACATGGTCC
