# Supplementary material for: Synergistic gene editing in human iPS cells via cell cycle and DNA repair modulation
Source: Nat Commun. 2020 Jun 8;11:2876. doi: 10.1038/s41467-020-16643-5 (PMC7280248; doi:10.1038/s41467-020-16643-5)
Supplement: Supplementary file 2 — Reporting Summary [file 41467_2020_16643_MOESM2_ESM.pdf]

nature research

Corresponding author(s): Knut Woltjen

Last updated by author(s): Apr 28, 2020

# Reporting Summary

Nature Research wishes to improve the reproducibility of the work that we publish. This form provides structure for consistency and transparency in reporting. For further information on Nature Research policies, see Authors & Referees and the Editorial Policy Checklist .

## Statistics

For all statistical analyses, confirm that the following items are present in the figure legend, table legend, main text, or Methods section.

| n/a | Confirmed | |
|---|---|---|
| ☐ | ☒ | The exact sample size (*n*) for each experimental group/condition, given as a discrete number and unit of measurement |
| ☐ | ☒ | A statement on whether measurements were taken from distinct samples or whether the same sample was measured repeatedly |
| ☒ | ☐ | The statistical test(s) used AND whether they are one- or two-sided<br>*Only common tests should be described solely by name; describe more complex techniques in the Methods section.* |
| ☒ | ☐ | A description of all covariates tested |
| ☒ | ☐ | A description of any assumptions or corrections, such as tests of normality and adjustment for multiple comparisons |
| ☐ | ☒ | A full description of the statistical parameters including central tendency (e.g. means) or other basic estimates (e.g. regression coefficient) AND variation (e.g. standard deviation) or associated estimates of uncertainty (e.g. confidence intervals) |
| ☒ | ☐ | For null hypothesis testing, the test statistic (e.g. *F*, *t*, *r*) with confidence intervals, effect sizes, degrees of freedom and *P* value noted<br>*Give P values as exact values whenever suitable.* |
| ☒ | ☐ | For Bayesian analysis, information on the choice of priors and Markov chain Monte Carlo settings |
| ☒ | ☐ | For hierarchical and complex designs, identification of the appropriate level for tests and full reporting of outcomes |
| ☒ | ☐ | Estimates of effect sizes (e.g. Cohen's *d*, Pearson's *r*), indicating how they were calculated |

*Our web collection on statistics for biologists contains articles on many of the points above.*

## Software and code

Policy information about availability of computer code

| | |
|---|---|
| Data collection | BD FACS Diva Software (LSR: v8.0.1, Aria II: v.8.0.2), Applied Biosystems 3130xl Genetic Analyzer Data collection software (v4.0) |
| Data analysis | FlowJo (v9.7.6 or higher), SnapGene (v5.0.2), TIDE (v2.01, https://tide.nki.nl/), Fiji (v1.51k or higher, ImageJ), LSM Software ZEN 2009, Microsoft Excel for Mac (v16.34) |

For manuscripts utilizing custom algorithms or software that are central to the research but not yet described in published literature, software must be made available to editors/reviewers. We strongly encourage code deposition in a community repository (e.g. GitHub). See the Nature Research guidelines for submitting code & software for further information.

## Data

Policy information about availability of data

All manuscripts must include a data availability statement. This statement should provide the following information, where applicable:

- Accession codes, unique identifiers, or web links for publicly available datasets
- A list of figures that have associated raw data
- A description of any restrictions on data availability

The data supporting the findings of this study are available from the corresponding author upon request. The source data underlying Figs 1-6 and Supplementary Figs 3-7 are provided as a Source Data file. The publicly available web tool TIDE (https://tide.nki.nl/) was used in this study.

# Field-specific reporting

Please select the one below that is the best fit for your research. If you are not sure, read the appropriate sections before making your selection.

# Life sciences study design

All studies must disclose on these points even when the disclosure is negative.

| | |
|---|---|
| Sample size | Two different iPS cell lines were used to exclude cell-line dependent effects. Multiple gene loci were used to reproduce the effect observed. |
| Data exclusions | No individual data points or outliers were excluded. Entire replicate data sets were excluded if controls did not fall within a standard range. |
| Replication | The data from at least two biological replicates was used. In some cases poor cell quality resulted in replicate data set exclusion. |
| Randomization | Randomization was not used as there were no human or animal participants and the study results could not be influenced by bias. |
| Blinding | Blinding was not used as there were no human participants and the study results could not be influenced by bias. |

# Reporting for specific materials, systems and methods

We require information from authors about some types of materials, experimental systems and methods used in many studies. Here, indicate whether each material, system or method listed is relevant to your study. If you are not sure if a list item applies to your research, read the appropriate section before selecting a response.

## Materials & experimental systems

| n/a | Involved in the study | |
|---|---|---|
| ☒ | ☐ | Antibodies |
| ☐ | ☒ | Eukaryotic cell lines |
| ☒ | ☐ | Palaeontology |
| ☒ | ☐ | Animals and other organisms |
| ☒ | ☐ | Human research participants |
| ☒ | ☐ | Clinical data |

## Methods

| n/a | Involved in the study | |
|---|---|---|
| ☒ | ☐ | ChIP-seq |
| ☐ | ☒ | Flow cytometry |
| ☒ | ☐ | MRI-based neuroimaging |

## Eukaryotic cell lines

Policy information about cell lines

| | |
|---|---|
| Cell line source(s) | 1383D6 human iPS cells (CiRA, Japan)<br>409B2 human iPS cells (CiRA, Japan) |
| Authentication | We performed SNP array for authentication of iPS cell lines.<br>These cell lines were also authenticated in the following references.<br>1383D6 human iPS cells (RIKEN BRC #HPS1006): Nakagawa M, et al. Scientific Reports 4: 3594 (2014)<br>409B2 human iPS cells (RIKEN BRC #HPS0076): Okita K, et al. Nat. Methods.8(5):409-12 (2011) |
| Mycoplasma contamination | All cell lines tested negative for Mycoplasma contamination. |
| Commonly misidentified lines<br>(See ICLAC register) | No commonly misidentified lines were used. |

## Flow Cytometry

### Plots

Confirm that:

☒ The axis labels state the marker and fluorochrome used (e.g. CD4-FITC).

☒ The axis scales are clearly visible. Include numbers along axes only for bottom left plot of group (a 'group' is an analysis of identical markers).

☒ All plots are contour plots with outliers or pseudocolor plots.

☒ A numerical value for number of cells or percentage (with statistics) is provided.

### Methodology

| | |
|---|---|
| Sample preparation | For measurement of GFP and BFP fluorescence intensities, 5x10^5 cells were resuspended in FACS buffer (PBS containing 2% FBS) and analyzed. For cell cycle analysis, cells were incubated with EdU (10µM), harvested, stained with Alexa Fluor 647 azide fluorescent dye, and then with PI (20µg/ml) before analysis. |

| | |
|---|---|
| Instrument | BD LSRFortessa Cell Analyzer<br>BD FACS ARIA II Cell Sorter |
| Software | Data collection: BD FACS Diva Software (LSR: v8.0.1, Aria II: v.8.0.2)<br>Data analysis: FlowJo software (v9.7.6 or higher) |
| Cell population abundance | Cell numbers were sufficient and normalized for each analysis. |
| Gating strategy | Preliminary FSC and SSC gates were set for doublet exclusion. |

☒ Tick this box to confirm that a figure exemplifying the gating strategy is provided in the Supplementary Information.
