# Supplementary material for: Synergistic gene editing in human iPS cells via cell cycle and DNA repair modulation
Source: Nat Commun. 2020 Jun 8;11:2876. doi: 10.1038/s41467-020-16643-5 (PMC7280248; doi:10.1038/s41467-020-16643-5)
Supplement: Supplementary file 3 — Peer Review File [file 41467_2020_16643_MOESM3_ESM.pdf]

Editorial Note: This manuscript has been previously reviewed at another journal that is not operating a transparent peer review scheme. This document only contains reviewer comments and rebuttal letters for versions considered at *Nature Communications*. Mentions of prior referee reports have been redacted.

Reviewers' Comments:

Reviewer #1:
Remarks to the Author:
Summary:
The authors have submitted a very commendable rebuttal to the Referee comments from their original submission, with satisfactory responses to most concerns. Most importantly, the authors have added additional data on endogenous sites, which was requested by all referees. Their results across 5/8 new endogenous sites aren't as impressive as the improvements in HDR rates observed across the original 2 sites, but overall the trend appears to constitute a meaningful advance. Pending that the authors address the remaining comments below, this Referee recommends publication.

Remaining comments:

1. In Figures 6g and 6h, 5/10 sites exhibit low levels of HDR (lower total editing + much lower HDR compared to the 5 other sites). Can the authors identify something about these sites, or the targeting strategy (distance of cut site from SNP) that would segregate these two groups of results?
2. Similar to comment #1, the effect of the cocktail appears to be most pronounced for sites/gRNAs with higher basal HDR. To make this point more clear, the authors should include a fold-change summary plots of the data in Figures 6g and 6h (dots for each site representing fold-change) so that readers can get a sense of the expected mean / range of improvement with no treatment, XL+N+S, and 32C+N+S.
3. Why do the authors observe a dichotomy of superior performance of XL+N+S or 32C+N+S depending on the site (split roughly evenly)?
4. Several Figure references in the rebuttal appear to refer to incorrect panels, making it difficult to follow the author's logic in their response.

Reviewer #2:
Remarks to the Author:
In the submitted manuscript, the authors described approaches to enhance HDR-mediated precise genome editing in pluripotent stem cells. The current work is a much improved version of their previous manuscript submitted to Nature Biotechnology. My biggest concern then was that the authors based their conclusions primarily on a traffic light reporter and that there was minimal validation on endogenous genomic loci. Hence, I am appreciative that the authors have tried to address this concern with additional experiments on a handful of endogenous target sites. Overall, the data look reasonably convincing. Below are some comments that I have for improving the authors' work.

1) Figure 4e: Why do the "-" (untreated control) and "DMSO" results look so different? The graph seems to suggest that the editing outcomes can be altered simply by adding DMSO (~12% vs ~30% HDR).

2) Figure 4g: Can the authors show DMSO, NU7441, and SCR7 in the graph as well?

3) Figure 6g-h: Ideally, there should be error bars with p-values to indicate whether any improvement is significant.

Best wishes,
Meng How Tan

Reviewer #3:
Remarks to the Author:
Highly efficient precise gene editing in stem cells remains a challenge. This manuscript describes a useful method to quantify HDR versus NHEJ in single cells during gene editing. Using a fluorescent reporter system, the authors can measure conversion of GFP to BFP using single cell FACS analysis to quantify the percentage of HDR versus NHEJ. Using different oligo donors, the manuscript show efficient derivation of monoallelic mutants using a number of technical improvements. The method will be a useful resource for precise gene editing. The observation of synergistic effects in the last figure is interesting.

Major comments:

1. The manuscript presents a number of technical advances, including a fluorescent reporter system, cell cycle control, modulating HDR/NHEJ pathways using small molecules, and cold shock conditions. I suggest they provide a final summary figure to system summarize what they learned, which is helpful for HDR in iPS cells and which is synergistic with other conditions. While several of these conditions are derived from prior works, a summary is helpful to the broad audience understand major observations from this work.

2. There are six main figures. Some of the figures can be moved to supplemental to keep the manuscript concise and easy to read.

3. I am interested in learning whether the conditions also altered cell growth, morphology, and viability. As a useful method, this information should be provided to readers for their evaluation.

4. The citation style is not for Nat Comm.

NCOMMS-20-10897-T

Synergistic gene editing in human iPS cells via cell cycle and DNA repair modulation

- Supplementary Figure 5d and 5e legends were replaced to match modifications made in the first revision.
- A correction was made in Supplementary Figure 6, changing “ssODN B” to “ssODN M”.
- Label annotation “Δ” was changed to “indel (Δ)” in Figure 4g and Supplementary Figures 6 and 7.

Reviewers’ comments:

Reviewer #1 (Remarks to the Author):

Summary:

The authors have submitted a very commendable rebuttal to the Referee comments from their original submission, with satisfactory responses to most concerns. Most importantly, the authors have added additional data on endogenous sites, which was requested by all referees. Their results across 5/8 new endogenous sites aren’t as impressive as the improvements in HDR rates observed across the original 2 sites, but overall the trend appears to constitute a meaningful advance. Pending that the authors address the remaining comments below, this Referee recommends publication.

We thank Reviewer #1 for acknowledging the meaningful advance of improved HDR rates with synergistic gene editing. We will address the remaining comments below.

Remaining comments:

1. In Figures 6g and 6h, 5/10 sites exhibit low levels of HDR (lower total editing + much lower HDR compared to the 5 other sites). Can the authors identify something about these sites, or the targeting strategy (distance of cut site from SNP) that would segregate these two groups of results?

We thank Reviewer #1 for their suggestion. HDR rates and total editing efficiency do not appear to be related to the distance of cut site from SNP, which were all within 6bp. One possible explanation for low editing rates is low on-target efficiency of the corresponding gRNA sequences. We predicted on-target gRNA efficiency using publicly available web tools. However, we found no common trend between predicted efficiency scores and our experimental data (Rebuttal Figures 1-3). Moreover, there were discrepancies in the scores for each gRNA between prediction tools. One experimental approach would be to test several gRNAs at each target site, where gRNAs with higher on-target efficiency would be expected to result in higher HDR. Still, this approach would not standardize other parameters, such as sequence context surrounding the break, which has also been shown to influence repair outcomes (PMID: 30405244).

DeepHF (http://www.deephf.com/index/#/Predict)

Target	Sequence	Score
KCNE1x255	TCTACATCGAGTCCGATGCC	0.375
KCNH2x134	GCCGTCATCTACTGCAACGA	0.675
SCN5Ax4282	GGACATTATGTATGCAGCTG	0.624

KCNH2x1763	ATCGGCTGGCTGCACAACCT	0.552
APRTx400	GGCAGCGTTCATGGTTCCTG	0.685
HES7x70	GCTTGTGGAGAAGCGGCGCC	0.522
PSMB8x601	ATAAGTGTTCCCACTACCCG	0.702
KCNJ11x878	GAAGGCGTGGTGGAAACCAC	0.717

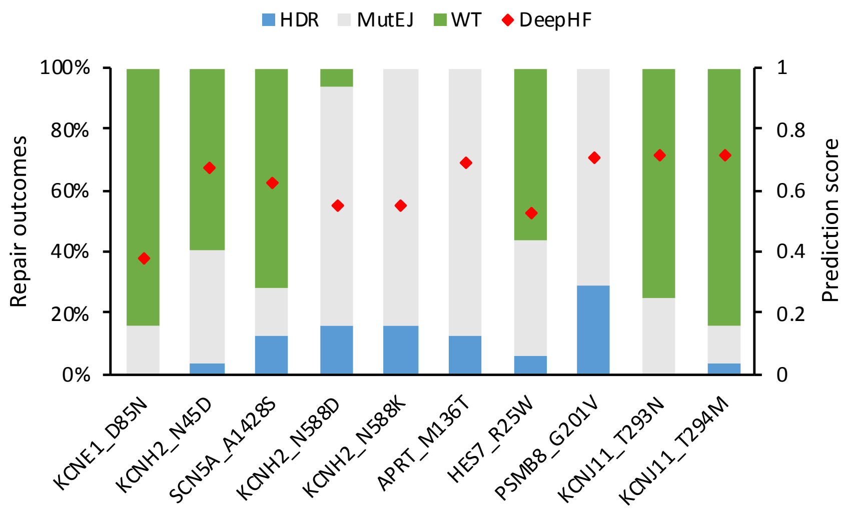

Rebuttal Fig. 1 DNA repair outcomes without treatment for all 10 targeting experiments from **Fig. 6g** and corresponding prediction scores with DeepHF (http://www.deephf.com/index/#/Predict).

CRISPOR "Fusi-score" (http://crispor.tefor.net/)

Target	Sequence	Score
KCNE1x255	TCTACATCGAGTCCGATGCC	58
KCNH2x134	GCCGTCATCTACTGCAACGA	69
SCN5Ax4282	GGACATTATGTATGCAGCTG	61
KCNH2x1763	ATCGGCTGGCTGCACAACCT	50
APRTx400	GGCAGCGTTCATGGTTCCTG	65
HES7x70	GCTTGTGGAGAAGCGGCGCC	46
PSMB8x601	ATAAGTGTTCCCACTACCCG	72
KCNJ11x878	GAAGGCGTGGTGGAAACCAC	57

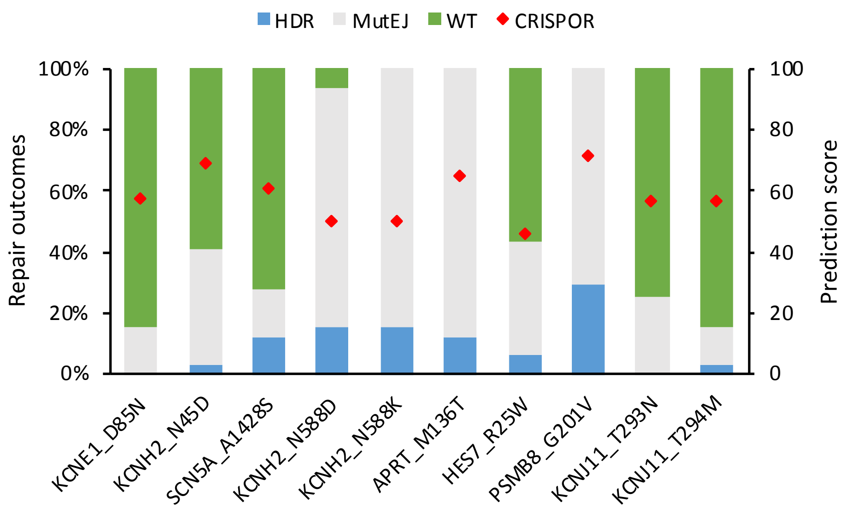

Rebuttal Fig. 2 DNA repair outcomes without treatment for all 10 targeting experiments from **Fig. 6g** and corresponding prediction scores with the CRISPOR gRNA efficiency prediction tool (http://crispor.tefor.net/). Marked scores correspond to "Fusi-scores" (PMID: 26780180).

CRISPR-Cas9 guide RNA design checker
(https://sg.idtdna.com/site/order/designtool/index/CRISPR_SEQUENCE)

Target	Sequence	Score
KCNE1x255	TCTACATCGAGTCCGATGCC	32
KCNH2x134	GCCGTCATCTACTGCAACGA	52
SCN5Ax4282	GGACATTATGTATGCAGCTG	37
KCNH2x1763	ATCGGCTGGCTGCACAACCT	61
APRTx400	GGCAGCGTTCATGGTTCCTG	26
HES7x70	GCTTGTGGAGAAGCGGCGCC	18
PSMB8x601	ATAAGTGTTCCCACTACCCG	60
KCNJ11x878	GAAGGCGTGGTGGAAACCAC	77

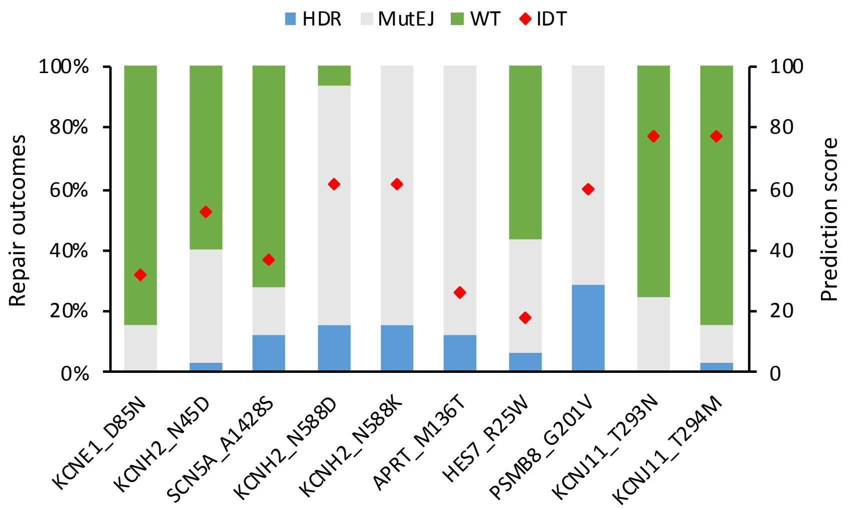

Rebuttal Fig. 3 DNA repair outcomes without treatment for all 10 targeting experiments from **Fig. 6g** and corresponding prediction scores with the IDT CRISPR-Cas9 guide RNA design checker tool (https://sg.idtdna.com/site/order/designtool/index/CRISPR_SEQUENCE).

2. Similar to comment #1, the effect of the cocktail appears to be most pronounced for sites/gRNAs with higher basal HDR. To make this point more clear, the authors should include a fold-change summary plots of the data in Figures 6g and 6h (dots for each site representing fold-change) so that readers can get a sense of the expected mean / range of improvement with no treatment, XL+N+S, and 32C+N+S.

Following the suggestion of Reviewer #1, a secondary axis was added in Figures 6g ang 6h to represent the fold change in HDR efficiency and HDR/MutEJ ratio with XL+N+S or 32°C+N+C treatments compared to basal HDR without treatment. The modified panels are shown below. While there seems to be a relationship between high overall editing and the effect of our modified conditions on HDR, higher basal HDR is not always correlated (e.g. HES7 versus PSMB8). We chose to not include the modifications in the revised manuscript.

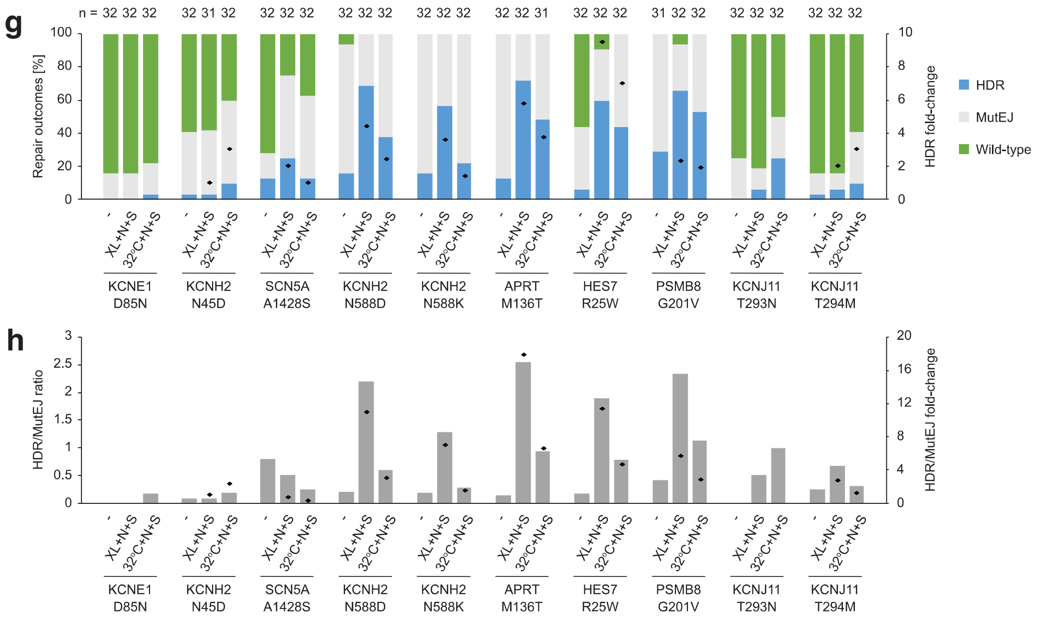

3. Why do the authors observe a dichotomy of superior performance of XL+N+S or 32C+N+S depending on the site (split roughly evenly)?

Given our results at the GFP reporter, we expected XL+N+S and 32C+N+S treatments to have a similar effect on HDR frequency. However, one or the other treatment showed higher performance depending on the target site. We consistently observed that XL+N+S treatment shows greater HDR improvement at high efficiency loci while 32°C+N+S treatment has a greater effect at low efficiency loci. This may indicate a non-cell cycle-dependent effect of cold shock, possibly by elongating RNP half-life and increasing the chance of cleavage at low efficiency loci. Non-cell cycle-dependent effects of cold shock on editing outcomes would be interesting to investigate in the future.

4. Several Figure references in the rebuttal appear to refer to incorrect panels, making it difficult to follow the author's logic in their response.

We apologize for any confusion resulting from incorrectly referenced panels. The rebuttal refers to panels from the initially submitted and revised manuscripts, which in some cases were changed.

Reviewer #2 (Remarks to the Author):

In the submitted manuscript, the authors described approaches to enhance HDR-mediated precise genome editing in pluripotent stem cells. The current work is a much improved version of their previous manuscript submitted to Nature Biotechnology. My biggest concern then was that the authors based their conclusions primarily on a traffic light reporter and that there was minimal validation on endogenous genomic loci. Hence, I am appreciative that the authors have tried to address this concern with additional experiments on a handful of endogenous target sites. Overall, the data look reasonably convincing. Below are some comments that I have for improving the authors' work.

We thank Reviewer #2 for positive remarks on the additional experiments performed on endogenous target sites. We will address the comments below.

1) Figure 4e: Why do the "-" (untreated control) and "DMSO" results look so different? The graph seems to suggest that the editing outcomes can be altered simply by adding DMSO (~12% vs ~30% HDR).

When individual DNA repair outcomes obtained using the homozygous GFP reporter line in Figure 4e were deconvolved in Figure 4g (see comment #2, below), the DMSO condition showed a high total editing efficiency in this experiment. The outcomes included a majority of biallelic indels (50.7%), followed by equivalent rates of HDR/indel (15.5%) and biallelic HDR (16.5%), and the lowest fraction of biallelic unmodified cells (12.5%) compared to other conditions. In this case, the overall high rate of total editing gives the impression that HDR is also improved.

2) Figure 4g: Can the authors show DMSO, NU7441, and SCR7 in the graph as well?

We prepared Figure 4g with all treatment conditions as shown below. However, as we felt that the additional data distracted from the purpose of the graph, we chose not to include the modifications in the revised manuscript.

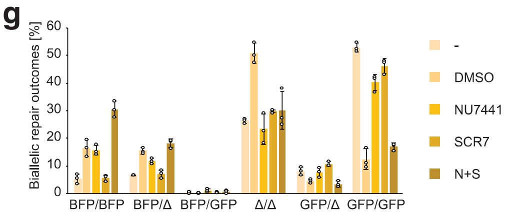

3) Figure 6g-h: Ideally, there should be error bars with p-values to indicate whether any improvement is significant.

In Figure 6g and 6h, we isolated single clones (n=32) for each treatment and endogenous target site rather than quantifying population data as was done at the GFP reporter. We felt that isolating and genotyping iPS cell clones would better represent the expected outcomes from practical targeting experiments, however does not lend itself to the statistical analysis suggested. Finally, source data for the figure will be provided as a Source Data file.

Reviewer #3 (Remarks to the Author):
Highly efficient precise gene editing in stem cells remains a challenge. This manuscript describes a useful method to quantify HDR versus NHEJ in single cells during gene editing. Using a fluorescent reporter system, the authors can measure conversion of GFP to BFP using single cell FACS analysis to quantify the percentage of HDR versus NHEJ. Using different oligo donors, the manuscript show efficient derivation of monoallelic mutants using a number of technical improvements. The method will be a useful resource for precise gene editing. The observation of synergistic effects in the last figure is interesting.

We thank Reviewer #3 for recognizing our advance for precise gene editing. We will address the remaining comments below.

Major comments:
1. The manuscript presents a number of technical advances, including a fluorescent reporter system, cell cycle control, modulating HDR/NHEJ pathways using small molecules, and cold shock conditions. I suggest they provide a final summary figure to system summarize what they learned, which is helpful for HDR in iPS cells and which is synergistic with other conditions. While several of these conditions are derived from prior works, a summary is helpful to the broad audience understand major observations from this work.

We thank Reviewer #3 for this helpful suggestion. We prepared a summary figure corresponding to the new Figure 7 in the manuscript.

2. There are six main figures. Some of the figures can be moved to supplemental to keep the manuscript concise and easy to read.

We also considering moving some of the main figures to supplemental as suggested, but concluded that each figure contains essential panels that are necessary to understand the conceptual and experimental process of this study.

3. I am interested in learning whether the conditions also altered cell growth, morphology, and viability. As a useful method, this information should be provided to readers for their evaluation.

Examples of how cell viability is affected by our experimental conditions are provided in Supplementary Figure 3h and the first rebuttal (response to Reviewer #1 comment #6), and cell viability from all experiments will be reported in the source data file. Conditions that result in cell cycle synchronization, such as cold shock and XL413 treatment visibly delay cell growth for the time of the treatment (24-48h), while no change in morphology was observed.

4. The citation style is not for Nat Comm.

We adjusted the citation style to Nat Comm in the revised manuscript.
